# Genomic Organization of the Newly Discovered Cassava Congo Cheravirus Reveals a Unique Maf/HAM1 Motif in the C-Terminal Region of the RNA1 Polyprotein and Suggests the Presence of Two Protein Domains Upstream of the Putative Helicase Domain

**DOI:** 10.3390/v18010084

**Published:** 2026-01-08

**Authors:** Yves Bisimwa Kwibuka, Stephan Winter, Espoir Basengere Bisimwa, Kumar Vasudevan, Hélène Sanfaçon, Hervé Vanderschuren, Sébastien Massart

**Affiliations:** 1 Plant Pathology Laboratory, TERRA-Gembloux Agro-Bio Tech, University of Liège, 5030 Gembloux, Belgium; 2 Department of Crop Production, Agriculture Sciences and Environment Faculty, Catholic University of Bukavu, Bukavu, Democratic Republic of the Congo; ebisimwa@yahoo.com; 3 Plant Virus Department, Leibniz Institute DSMZ-German Collection of Microorganisms and Cell Cultures, 38124 Braunschweig, Germany; stephan.winter@dsmz.de; 4 Plant Genetics and Rhizospheric Processes Laboratory, TERRA-Gembloux Agro-Bio Tech, University of Liège, 5030 Gembloux, Belgium; kvasudevan@uliege.be (K.V.); herve.vanderschuren@uliege.be (H.V.); 5 Laboratory of Tropical Crop Improvement, Division of Crop Biotechnics, Biosystems Department, KU Leuven, 3000 Leuven, Belgium; 6 Summerland Research and Development Centre, Agriculture and Agri-Food Canada, Summerland V0H 1Z0, BC, Canada; helene.sanfacon@agr.gc.ca

**Keywords:** cassava viruses, cheravirus diversity, emerging plant viruses, HAM/ITPase domain, molecular epidemiology, recombination events, protein cleavage sites, secovirid polyprotein

## Abstract

Cassava (*Manihot esculenta*) is a staple crop in sub-Saharan Africa threatened by several viral diseases. Here, we describe the genome sequence of a novel bipartite cheravirus (family *Secoviridae*) infecting cassava in the Democratic Republic of Congo and Tanzania. We designate the new virus “cassava Congo cheravirus”. Each RNA segment encodes a single polyprotein (P1 and P2 for RNA1 and RNA2, respectively), embedded with various putative cleavage sites (six and three in P1 and P2, respectively), consistent with members of the genus *Cheravirus*. We note two new features in the P1: (i) the presence of two domains, X1 and X2, upstream of the putative helicase region, which we also predict in other cheraviruses and (ii) the presence of a Maf/HAM1-like inosine triphosphatase (ITPase) domain, a rare motif among viruses only previously detected in three potyviruses and a torradovirus, all of which infect plants from the Euphorbia family. Phylogenetic analyses placed the virus firmly within the genus *Cheravirus*, with amino acid identities in the Pro-Pol and coat protein regions well below existing ICTV species thresholds, supporting its classification as a virus belonging to a new species in the *Cheravirus* genus. Spatially distinct isolates from Bas-Congo, South-Kivu, and Tanzania form three genetic clusters, with evidence of recombination in both RNA segments. These results expand the known diversity of cassava viruses and suggest possible adaptation to the cassava host via ITPase acquisition.

## 1. Introduction

In several countries worldwide, the daily caloric needs of millions of people are met through cassava-based foods. The importance of cassava for food security and income is high in sub-Saharan Africa due to its ability to yield storage roots and produce reasonable yields in poor soils with minimal inputs [1]. The threats posed by climate change are increasing the magnitude of the daily challenges facing African agriculture, food security, and nutrition [2]. In this particular context, cassava is recognized as a crop on which African farmers could rely in the future, compared to other leading crops in sub-Saharan Africa (SSA) [3]. The main factors on which this reputation of cassava as a “food bank for the poor” is based include its capacity to grow under environmentally challenging conditions (e.g., drought, low fertility, high temperatures) [4,5,6].

One of the most important limitations for cassava production in Africa is its susceptibility to various pathogens, arthropods and mammal pests [7]. The primary factors that facilitate disease establishment and spread are the vegetative propagation of the crop, the presence of virus vectors, and the long vegetation period that helps to maintain pathogens and pests. From the long list of known pathogens affecting cassava [4,8], *Xanthomonas axonopodis* pv. *manihoti*, the cassava brown streak viruses (CBSVs) and the cassava mosaic viruses (CMVs) [1,9,10,11,12] cause the most economic impact. In East and Central Africa, the two virus species associated with cassava brown streak disease (CBSD) and the nine virus species associated with cassava mosaic disease (CMD) are the most important pathogens causing severe yield losses [13]. The list of potentially threatening pathogens is continually expanding with the discovery of new viruses infecting cassava in Africa [8] and America [14,15].

Here, we present the biological and molecular characteristics of a new bipartite virus from the family *Secoviridae*, detected and isolated from cassava plants grown in the Democratic Republic of the Congo (D.R. Congo).

*Secoviridae* is the only family of plant-infecting viruses [16,17] in the order picornavirales. The viruses in the family share common properties with other members of the order: (a) a linear positive-strand RNA genome which can be monopartite or bipartite, with each RNA flanked at its 5′ end by a small viral protein, the “VPg”; (b) the presence of a large ORF that encodes a single large polyprotein cleaved by a 3C-like cysteine protease; (c) a conserved “replication bloc” within the RNA1-encoded polyprotein that includes a type III helicase (also referred to as NTP-binding protein or NTB), the VPg, a cysteine protease and a type I RNA-dependent RNA-polymerase; (d) a capsid, composed of coat protein (CP) subunits, that can be divided into a single large CP, or into two or three smaller CPs depending on the genus [18,19,20]. To date, 165 virus species belonging to one subfamily, ten genera and five subgenera have been assigned to the family *Secoviridae* (ICTV online taxonomy release, 22 August 2025). The genus *Cheravirus* comprises viruses with a bipartite genome that encodes three capsid proteins on RNA2. RNA1 encodes replication proteins and the protease. Cheraviruses are transmitted by nematodes and seeds [18]. Most cheraviruses can also be transmissible experimentally by mechanical inoculation [18]. Five criteria, not to be simultaneously met, are used to separate genera within the family *Secoviridae,* whereas seven are helpful in the demarcation of species: a CP amino acid sequence with less than 75% identity, a conserved amino acid sequence region located between the protease and the polymerase domains (Pro-Pol) with less than 80% identity, differences in antigenic reactions, distinct host range, distinct vector specificity, absence of cross-protection and absence of re-assortment between RNA1 and RNA2 for viruses with a bipartite genome [18].

The unique feature of this new secovirus isolate from cassava is the presence of a Ham1 gene in its genome. Maf1/ham1-like proteins (HAM1) are ubiquitous proteins among all living organisms, with a pyrophosphatase activity (ITPase, also termed the “house-cleaning” activity) to detoxify harmful non-canonical nucleotides: inosine triphosphate (ITP), xanthosine triphosphate (XTP) and their deoxy analogues (dITP/dXTP) [21,22,23]. Until recently, only three viruses, all from the *Potyviridae* family, were known to encode a HAM1 protein homolog to ITPases: CBSV and UCBSV (genus *Ipomovirus*) [24] and Euphorbia ringspot virus (EuRSV, genus *Potyvirus*) [25]. Recently, the cassava torrado-like virus (CsTLV, genus *TORRADOVIRUS*) of the *Secoviridae* family has also been shown to encode a HAM1-like protein [15,26,27]. Interestingly, all these viruses were isolated from euphorbiaceous host plants (cassava and the ornamental plant *Euphorbia milii*), for which previous studies have hypothesized higher concentrations of ITP/XTP in the cytoplasm due to the relocation of the plant HAM1 into the nucleus [21,24,28,29]. This high cytoplasmic pool of ITP may act as a selection pressure, particularly on cytoplasm-localized viruses, and promote the acquisition of the HAM1 gene in the viral genome to enable their survival in euphorbiaceous hosts [30].

## 2. Materials and Methods

### 2.1. Virus Isolation from Cassava Collected in Bas-Congo

**Figure 1:**
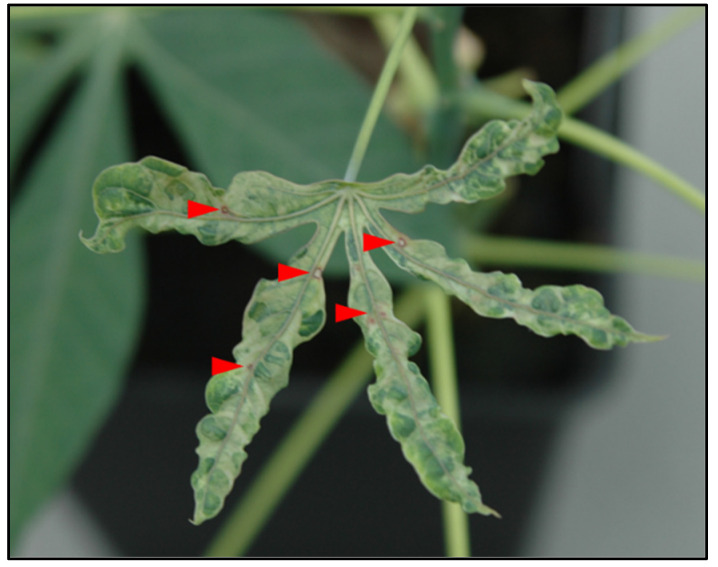
Cassava plant collected in 2007 from stem cuttings and maintained in the DSMZ greenhouse at DSMZ. Red necrotic spots (indicated by red arrows) and severe mosaic symptoms suggest the presence of a virus or other pathogen.

In 2007, during field studies on the diversity of CMD conducted in Democratic Republic of the Congo (Bas-Congo), samples (stem cuttings) were collected from cassava plants. Cuttings were rooted, and plants were established and maintained under greenhouse conditions at the Plant Virus Collection, Leibniz-Institute German Collection of Microorganisms and Cell Cultures (DSMZ). Leaves from virus-infected cuttings exhibited typical mosaic symptoms; however, one cassava plant showed severe mosaic symptoms, leaf blistering and deformation, and conspicuous red necrotic spots, indicating the presence of additional viruses or other pathogens (Figure 1).

Leaf extracts were prepared from leaves of infected plants and mechanically inoculated to a set of herbaceous virus indicator hosts from the families Cucurbitaceae, Solanaceae and Chenopodiaceae, including *Nicotiana occidentalis* and *N. benthamiana.* For electron microscopy, sap extracts from symptomatic *N. benthamiana* leaves were adsorbed onto copper grids (400 mesh × 62 μm pitch), negatively stained with uranyl acetate, and examined by electron microscopy (EM).

For virus purification, leaves from virus-infected *N. occidentalis* plants were homogenized 10 days post-infection and subsequently subjected to differential centrifugation, including density gradient centrifugation, essentially following a protocol originally designed for tomato ringspot virus [31]. An antiserum against purified virus particles was then prepared (DSMZ AS-0896).

Soluble leaf proteins from virus-infected *N. benthamiana* plants and purified particle preparations were subjected to a discontinuous sodium dodecyl sulfate-polyacrylamide gel electrophoresis (SDS-PAGE, gradient 8–20%) to determine the size of the viral coat protein. Following electrophoresis and transfer to nitrocellulose membranes, viral proteins were detected by incubating the membranes with the antiserum DSMZ AS-0896.

Purified virus preparations were subjected to cDNA synthesis using random-primed cDNA synthesis followed by 2nd strand synthesis and cloning into a pBluescript SK^-^ cloning vector. Positive clones from blue-white screening were sequenced with vector-specific primers, and contigs were assembled using Vector NTI 10.5. PCRs spanning missing genome sequences and 5′ and 3′ RACEs (as described below) were performed to reconstruct the complete viral RNA1 (7405 nt) and RNA2 (3460 nt) genomes. Genome sequences for this virus isolate PV-0896 were deposited into GenBank (RNA1, ON_924323; RNA2, ON_924324) and were included in the molecular analysis and diversity study of cassava virus samples collected in South Kivu Province, analyzed by HTS.

### 2.2. Virus Survey in South Kivu Province

#### 2.2.1. Sampling, Pooling and Greenhouse Cultivation

From April to October 2019, during investigations on the diversity of viruses present in South Kivu/Eastern Democratic Republic of the Congo, samples were collected in the Uvira territory as previously indicated [32]. Surveyed fields in each village were randomly selected along transects oriented within the main cassava-growing villages, with a minimum distance of 2 km between fields. Two hundred forty-two fields were sampled in total. In each field, a portion of the third fully expanded young leaf on a shoot was collected on ten cassava plants selected following diagonals. Samples from individual plants, symptomatic or not, were conserved in tubes dried with silica gel. All leaf samples from the same field were pooled together to make a unique field-core sample. Stem cuttings from each sampled plant were also collected and grown in an experimental field in Bukavu, while others were shipped to Belgium and grown in a glasshouse at 27 °C.

**Figure 2:**
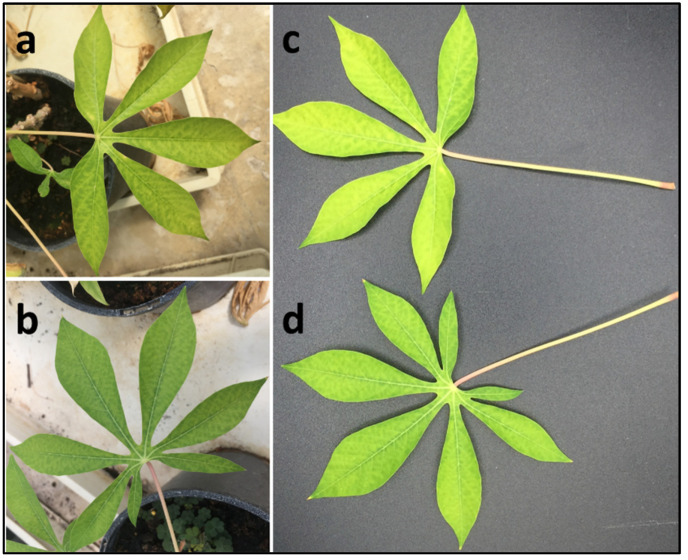
Cassava landraces ((**a**): Nabwilalanga, (**b**): Nabwigoma, (**c**,**d**): Rava) collected in 2019 as stem cuttings and grown in a greenhouse at Gembloux Agro Bio-tech, showing pale and irregular chlorotic mottle pattern of leaves.

Cassava plants regenerated through stem cuttings, kept in a glasshouse (27 °C) showed a chlorotic mottle pattern on middle leaves, consisting of irregular, pale interveinal yellow-green patches scattered across the lamina, spreading from secondary veins (Figure 2). These symptoms were absent on newly expanded leaves but developed on mature leaves. The plants tested negative for CBSVs [32] and *Manihot esculenta*-associated ampelovirus (MEaV-1 and MEaV-2) [8] by RT-PCR.

#### 2.2.2. RNA Extraction, Pooling of Field Samples, and High-Throughput Sequencing

Total RNA was extracted from each field sample (*n* = 242) using a modified CTAB protocol as previously described [8]. Extracted RNA was further tested for infection with cassava brown streak virus (CBSV) and Ugandan cassava brown streak virus (UCBSV) by RT-PCR, as previously described [32].

All samples that tested negative with this primer-based screening (71 field-core samples representing 710 individual plants collected into 71 farmer fields) were regrouped into 12 equimolar HTS pools (Pool number 1 to Pool number 12) that were subjected to high-throughput sequencing (HTS). DNase treatment was performed using Amplification-grade DNase I according to the manufacturer’s instructions (Life Technologies, Carlsbad, CA, USA). In addition, the RiboZero plant leaf kit for RNA-seq (Life Technologies Limited, Paisley, UK) was used for depleting ribosomal RNAs, and a TruSeq stranded total RNA kit (Illumina, New York, NY, USA) was used for library preparation following the manufacturer’s instructions. RNA libraries were then subjected to NextSeq 500 HTS at the University of Liège GIGA facilities (Liège, Belgium), with a read length of 2 × 75 bp in high output mode. Each library (pool) was sequenced at 5 million reads at 30× depth. In total, approximately 60 million reads were generated. All sequenced read data were deposited in the Sequence Read Archive (SRA) under the bioproject reference PRJNA1311516. Detailed SRA accessions for each sequenced pool are provided in Table S1.

#### 2.2.3. Bioinformatic Analyses

Sequence reconstruction and analysis were performed using Geneious 2021.1.1 software (www.geneious.com) and embedded plugins. Following demultiplexing and quality trimming (BBDUCK, BBMerge, and Dedupe plugins), reads were de novo assembled into contigs using RNA-SPADES (embedded in Geneious). Reconstructed contigs were screened against the RefSeq non-redundant viral database (release 202, 8 September 2020, retrieved from NCBI) using BLASTn and tBLASTx searches (version 2.10.1) [33]. Early screening of reconstructed contigs from 4 HTS pools (Pool number 3, Pool number 4, Pool number 5, Pool number 12) against the RefSeq Viral database revealed nucleotide sequence identities with members of the family *Secoviridae*. Twenty-five rounds of iterative read mapping extended the size of corresponding contigs to yield the final sequences.

Multiple sequence alignments were reconstructed using ClustalW embedded in Mega X [34]. Maximum-likelihood phylogenetic trees were reconstructed in MEGA X after selecting the appropriate model for amino acid or nucleotide sequence alignments. The Le Gascuel (LG) matrix-based model of amino acid substitution using a discrete Gamma distribution with invariant sites (+G+I) was used on the alignment of the conserved amino acid “Pro-Pol” region, which is defined as the region between the conserved CG motif of the protease and GDD motif of the polymerase (in the RNA1 polyprotein). In contrast, the same substitution matrix (LG), but with frequencies and a discrete Gamma distribution (+G+F), was used to align the coat protein amino acid sequences (in the RNA2 polyprotein). These trees were constructed using translated amino acid sequences derived from nucleotide sequences of the RNA1- and RNA2-encoded polyproteins for reconstructed genomes and from all available genomes of *Secoviridae* members retrieved from GenBank. Table S2 lists the names of these viruses and the accession numbers for their corresponding RNA1 and RNA2 genomes. Bootstrap analysis (1000 replicates) was performed to evaluate the stability and significance of branches.

Putative reassortments and recombination events within the RNA segments were investigated using the methods implemented by the RDP4 software (version Beta 4.97). Recombination events supported by more than four of the nine integrated methods were considered as possible [35].

#### 2.2.4. Confirmatory RT-PCR, Rapid Amplification of cDNA Ends (RACE), and Sanger Sequencing

Primers were designed to confirm the presence of viral sequences in pooled samples from multiple fields and in individual samples. Primers were also designed to reconstruct the 5′ and the 3′ ends of the assembled genomes. Additional primers were also designed to confirm the presence of the identified HAM1 motif in the RNA1 polyprotein by amplifying the region spanning the motif. All primers are shown in Table S3.

For confirmation of virus presence in pools of individual fields, RNA was extracted using the RNEasy Plus^®^ Kit (QIAGEN^®^, Hilden, Germany) following the manufacturer’s recommendations. For the RT-PCR confirmation, the first-strand cDNA synthesis was performed using Tetro™ Reverse Transcriptase (Meridian Bioscience, Luckenwalde/Berlin, Germany) and random hexamers, following the manufacturer’s instructions. The PCR was conducted using Mango*Taq*™ DNA polymerase (Meridian Bioscience). Primer pairs were designed to amplify both extremities (5′ and 3′) of RNA1 and RNA2 (Table S3). A 10 µL PCR mixture containing 5.8 µL nuclease-free water, 2 µL PCR buffer (5X), 0.40 µL MgCl_2_ (50 mM), 0.20 µL dNTPs (10 mM), 0.20 µL of each primer (10 mM), 0.4 µL Mango Taq DNA polymerase (Meridian Bioscience^®^, Luckenwalde/Berlin, Germany), and 1.0 µL of cDNA. PCR products were analyzed by electrophoresis in TAE buffer (1X) on a 1% agarose gel stained with Gel red^®^ (Biotium, Fremont, CA, USA), visualized under UV light, and photographed using a gel documentation system (E-Box CX5 Edge, Vilber/Fisher Biotech, Paris, France).

Reconstruction of the 5′ and 3′ ends of genomes was conducted using the SMARTer^®^ RACE 5′/3′ Kit (Takara Bio, San Jose, CA, USA) following the manufacturer’s instructions. Obtained RACE products were Sanger-sequenced using virus-specific primers (Table S3).

### 2.3. Identification of the RNA1 and RNA2-Encoded Cleavage Sites

Nucleotide sequences corresponding to the RNA1 and RNA2 encoded polyproteins of genomes reconstructed in this study, together with those of representative members of the *Secoviridae* family (Table S4), were translated into amino acid sequences and aligned using Clustal Omega (Version 1.2.4) [36]. These alignments were used to deduce functional domains based on sequence homologies with related viruses. Putative cleavage sites were then inferred from alignments of cassava Congo cheravirus polyproteins (P1 and P2, respectively) with related *Secoviridae* members for which protease cleavage sites are experimentally characterized [37,38]. All isolates carried the conserved substrate-binding histidine in the protease domain (Figure S1), typically associated with cleavage specificity at Q or E in the −1 position, as shown for picornaviruses [39], and several members of the family *Secoviridae*, including cheraviruses [16]. However, in the case of nepoviruses, some proteases with the histidine in the substrate-binding pocket also recognize N, D or H at the −1 position [40].We therefore searched for putative cleavage sites that included Q, E, N, D or H in that position.

### 2.4. Data Mining

To screen for the newly identified *Secoviridae* members in publicly available RNASeq data, the nucleotide sequences corresponding to the polymerase domain encoded into the RNA1 segment of the secovirids detected in field-grown cassava plants were screened against the SRA [41] using an RdRp-based search. This search uses the core of the polymerase palm subdomain [42]. The Serratus cloud computing infrastructure was used to implement this search [43]. Raw reads data identified by the Serratus search as containing palm-id motifs closely related to the RdRp of the novel *Secoviridae* members were retrieved from the SRA using the “Faster Download and Extract Reads in FASTQ format from NCBI SRA” tool integrated into the GALAXY Europe platform [44]. The reads were processed following the bioinformatic analyses detailed in Section 2.2.3 above, using the pipeline and tools stated above. The contigs showing homology to *Secoviridae* were generated and included in subsequent analyses.

All generated contigs were deposited in Genbank and assigned accession numbers shown in Table 1.

## 3. Results

### 3.1. Isolation, Electron Microscopy, Purification, and Complete Genome Reconstruction of a Cheravirus Isolate from Bas-Congo

**Figure 3:**
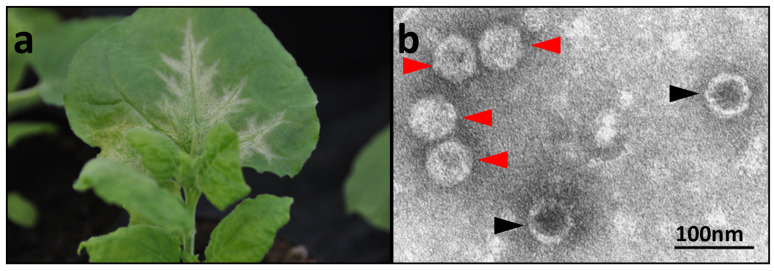
(**a**) Virus symptoms in *N. benthamiana* starting with chlorotic mottle and deformation of the youngest leaves. (**b**) Electron micrograph of leaf homogenates showing VLp of icosahedral structure (red arrows), sometimes empty (black arrows).

*N. benthamiana* plants infected by mechanical inoculation started showing chlorotic mottle and deformation of the youngest leaves approximately 15 days after inoculation, followed by vein clearing and oak leaf patterns developing on older leaves (Figure 3a). Electron microscopical examinations revealed virus-like particles (VLps) of icosahedral structure (Figure 3b, red arrows), some of which were penetrated by uranyl acetate stain, indicating that they were empty protein shells (Figure 3b, black arrows).

**Figure 4:**
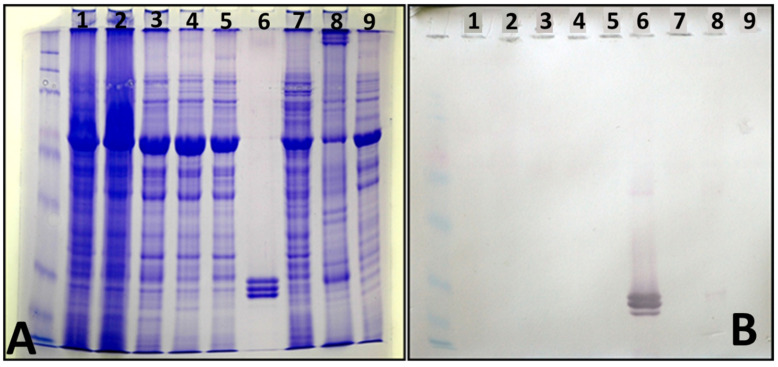
(**A**). Electrophoretic separation (8–20% SDS-PAGE) of soluble leaf proteins from infected *N. benthamiana* (lanes 1–5, 7, and 8) and purified virus preparation (lane 6). (**B**), Nitrocellulose western immunoblot incubated with the antiserum DSMZ AS-0896, indicating virus coat protein units (lane 6).

Electrophoresis in SDS-PAGE revealed three protein bands in purified virus preparations (Figure 4A, lane 6), indicating the presence of three putative coat protein units of sizes varying between 20 and 30 kDa (Figure 4A). Western immunoblot analysis with the virus-specific antiserum confirmed this finding (Figure 4B, lane 6). Also, it showed a faint reaction with protein preparations from virus-infected *N. benthamiana* (Figure 4B, lane 8).

Results from the complete genome assembly revealed that RNA1 and RNA2 are 7405 and 3460 nt long, respectively (Table 1). The presence of three coat proteins and the bipartite nature of the virus genome is typical of members of the genus *Cheravirus.* The genome sequence of the virus isolate designated CGO-BC, which serves as the reference sequence, has been submitted to GenBank (see below).

### 3.2. Genome Sequences of the New Cheravirus from Eastern Democratic Republic of the Congo and Data Mining of Publicly Available RNASeq Data

Annotation of contigs assembled from field-grown cassava plants in South Kivu identified eight contigs that showed high nucleotide and amino acid sequence homology with the genome sequence of the cheravirus isolate from Bas-Congo. These contigs originated from four of the twelve sequenced HTS pools (Pool 3, 4, 5 and 12—Table S1).

To uncover other secoviruses genome sequences from the publicly available RNASeq data, the polymerase sequence of the CGO-BC isolate was used for an RdRp search using the Serratus tool [43]. This data screening identified >80% identity with an RdRp palm_id reference u138608 from the RNASeq ERR996012 generated in 2015 to detect and assemble the genomes of CBSV and UCBSV [45]. The corresponding RNASeq dataset was downloaded, and, following demultiplexing, de novo assembly, and BLAST (version 2.10.1) searches as specified above, three contigs showing homology to secovirids were retrieved.

The sequence annotation of the 11 reconstructed contigs via BLASTn on nucleotide database revealed that in total, two groups could be defined according to their sizes: one group of five long contigs (four from field sampling and one from data mining) with sizes varying between 7405 nt and 7795 nt, showing distant similarities with the RNA1 of the stocky prune virus (YP_009665965.1), and another group of six shorter contigs (four from field sampling and two from data mining) with sizes varying between 3460 and 3496 nt (Table 1) showing distant homologies with RNA2 of the same virus (YP_009665964.1).

For the genome sequences identified in South Kivu province and through data mining in Tanzania, the CGO-SK and TZ initials were used, respectively. All 3′ ends were successfully retrieved by 3′ RACE (Note that the contigs retrieved through the SRA RNASeq data were not included in the RACE assay). Alignment of the TZ-55 contig with the CGO-BC reference suggested that the complete 5′ end was recovered. The 11 complete or nearly complete genome sequences were annotated and submitted to NCBI (reference numbers in Table S1). On the other hand, results from field-grown plants also indicated the presence of begomovirus-associated satellites II and III in one pool (Pool 4) and UCBSV in two pools (4 and 5) (accession numbers MW961202.1, MZ486425.1, MW961214.1, MW961221.1). Using publicly available RNASeq data, the results also allowed the identification of both CBSV (KR108830.1, 100% id) and UCBSV (KR108837.1, 100% id) in the same read run in which the secovirid contigs were identified (ERR996012).

### 3.3. Genome Annotation and Phylogenetic Analysis

#### 3.3.1. Genome Sequences and Annotation

Each of the 13 genome segments showed the canonical organization of cheraviruses, with RNA1 and RNA2 each encoding a single polyprotein. The RNA1 polyproteins ranged from 2369 to 2483 aa, while RNA2 polyproteins ranged from 1067 to 1069 aa (Table 1). The size of the 5′ and 3′UTR regions of the reference isolate (CGO-BC) was 131 and 139 nucleotides for RNA1 and 95 and 139 nt for RNA2. The length of 3′UTR for RNA1 was 153 or 154 nucleotides for South Kivu isolates (fully sequenced) and only 120 nt for the Tanzania isolate (partial sequence). For RNA2, the length of 3′UTR ranged between 150 and 153 nt for South Kivu isolates (fully sequenced). A similar size (153 and 158 nt) was observed for the two RNA2 sequences from Tanzania, but the polyA tail was not reached. All 13 generated contigs were deposited in GenBank and assigned accession numbers shown in Table 1.

#### 3.3.2. Phylogenetic Analysis

Pairwise amino acid comparisons and phylogenetic reconstructions were conducted using two regions: (i) the conserved protease-to-polymerase domain (the Pro-Pol region between the “CG” motif of the protease and the “GDD” motif of the polymerase) of RNA1, and (ii) the coat protein (CP) sequences. The 13 genomes of the putative new cheravirus were included, as well as 63 genomes of other representative *Secoviridae* species (see Table S2).

**Figure 5:**
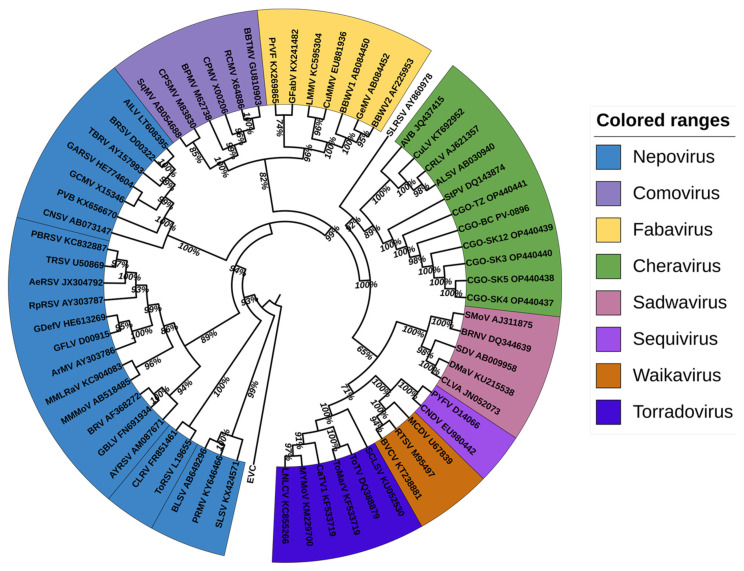
Phylogenetic analysis of the aligned Pro-Pol amino acid region (between the “CG” motif of the protease and the “GDD” motif of the polymerase) of the cassava Congo cheravirus isolates (highlighted in green together with members from the cheravirus genus) and of the exemplar isolates from recognized species in the family *Secoviridae* (detailed information on these viruses is provided in Table S2). The amino acid sequence of the “Pro-Pol” region was deduced from the nucleotide sequence of the corresponding genomic RNA from the representative virus of each genus in the *Secoviridae* family. The alignments were generated using ClustalW integrated into MEGA X. The Maximum likelihood phylogenetic trees were also reconstructed in MEGA X using the Poisson model with uniform distribution for amino acid sequence alignments. The trees were annotated and formatted using iTOL v6 [46]. Bootstrap values are displayed at internal branch nodes and expressed as percentages based on 1000 replicates. The tree was rooted using the Pro-Pol sequence of poliovirus (EVC, species *Enterovirus C*, genus *Enterovirus*, family *Picornaviridae*).

Both analyses yielded congruent topologies; the Pro-Pol-based tree is presented in Figure 5 and the CP-based tree in Figure S2.

The cassava isolates consistently clustered with recognized members of the genus *Cheravirus,* including stocky prune virus (StPV), arracacha virus B (AVB), currant latent virus (CuLV), cherry rasp leaf virus (CRLV), and apple latent spherical virus (ALSV) with strong bootstrap support.

Amino acid sequence identities between the cassava isolates and known *Secoviridae* species members were below 47% for the Pro-Pol region and below 13% for the CP region (Table 2). These values fall well below the ICTV species demarcation thresholds of 80% (Pro-Pol) and 75% (CP), supporting the classification of these isolates as a novel *Cheravirus* species. The Pro-Pol region of all isolates described for the new cheravirus exhibited a 36-aa insertion, as shown in Figure S3.

### 3.4. Genomic Diversity of the Viruses Included in the New Cheravirus Species

Pairwise amino acid sequence identities amongst isolates of the new virus indicated that the lowest identity percentage for the Pro-Pol and CP sequences were 84% (BC and TZ isolates) and 70% (SK and TZ isolates), respectively (Table 3). The isolates from South Kivu showed high sequence identities, with a minimum of 96.5% and 97.6% for Pro-Pol and CP, respectively.

Phylogenetic reconstructions based on these alignments (and on the complete genome sequences) revealed that the 4 isolates from South Kivu formed a distinct cluster regardless of the genomic region considered. In addition, for RNA2, the two Tanzanian isolates always clustered together (Figure S4). The phylogenetic placement of the Bas-Congo reference isolate (CGO-BC) varied depending on the genomic region analyzed: clustering with South-Kivu (Pro-Pol and Polyprotein 1), with Tanzania (CP and RNA1 genome), or in a single cluster (Polyprotein 2 and RNA2 genome), suggesting possible reassortment or recombination of the RNAs.

Recombination analysis (RDP4) suggested recombination events in both RNA1 and RNA2, with several events supported by at least four algorithms. For example, CGO-SK3 and CGO-SK5 were identified as recombinants in RNA1 (the Pro-Pol region comprised between nucleotides 189 and 406 of the reference genome), while CGO-SK12 and TZ55 displayed recombination features in RNA2 (nucleotides 1 to 328) (Table S5).

Given that the ICTV demarcation criteria for amino acid identity (Pro-Pol < 80% and CP < 75%) were not consistently met across comparisons, we propose that all cassava isolates described here represent a single novel species within the genus *Cheravirus*. We suggest the name “cassava Congo cheravirus” for this novel virus. Further separation into additional species may require biological evidence, such as host range, vector specificity, or re-assortment capacity [18].

### 3.5. Prevalence of the Newly Characterized Cheravirus in South Kivu Province

**Figure 6:**
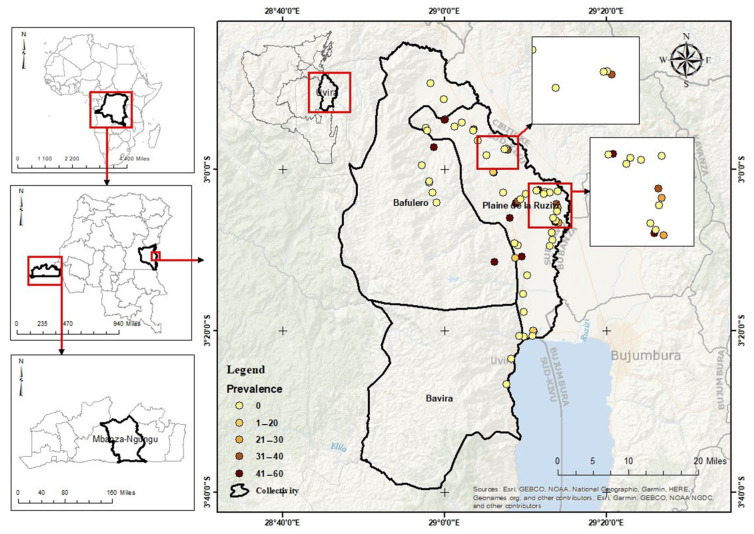
Geographical distribution and prevalence of cassava Congo cheravirus in the 71 cassava fields of Uvira territory in the South Kivu province/Democratic Republic of the Congo. The central map shows the surveyed territory, with its three collectivities (Bafulero, Bavira, and Plaine de la Ruzizi). A dot represents each surveyed field. Yellow dots correspond to negative fields, while orange and dark red dots indicate positive fields with increasing prevalence. The panels on the left locate the study area at the national and continental scales.

Using HTS analyses of pooled samples, the new cheravirus was detected in 4 pools, including 33 fields, whereas the 8 other pools (representing 38 fields) tested negative (no viral reads). Among the four pools, RT-PCR testing of field-core samples indicated that 23 fields were infected with the virus. The field prevalence was therefore 32% (23 of 71 sequenced). The individual samples were also tested by RT-PCR, yielding 130 positive results among the 230 tested plants. The incidence within an infected field ranged from 20 to 60% with an average of 40%. The geographical repartition of infected and non-infected fields is illustrated in Figure 6.

The highest proportion of positive fields with higher prevalence (30–60%) clustered around the “Plaine de la Ruzizi” collectivity, located in the North-Western part close to Burundi and Rwanda, while southern and eastern areas showed mostly negative or low-prevalence detections (0–20%), suggesting either lower virus circulation or undersampling. These negative fields are interspersed with positive fields, indicating that infection is not uniform within hotspot-like zones, likely due to the presence of landraces that may not have been inoculated or that did not originate from virus-carrying material. Hotspots could be linked to intensive cassava cultivation, movement of planting material, or favorable agroecological conditions for virus persistence. This patchiness suggests localized spread rather than territory-wide prevalence.

### 3.6. Identification of the RNA1 and RNA2 Encoded Proteins and Cleavage Sites

**Figure 7:**
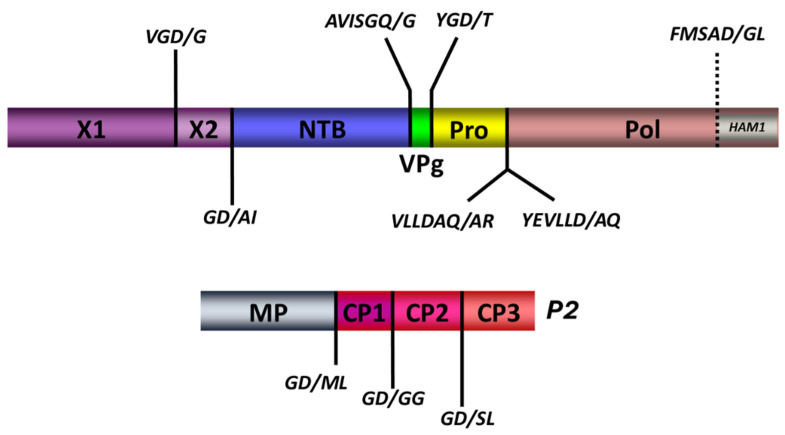
Putative consensus cleavage sites delineating putative functional domains predicted within the RNA1 (P1) and the RNA2 (P2)-encoded polyproteins. Predicted cleavage sites are shown as short vertical lines above and behind the full-length polyproteins, along with the predicted cleaved peptide in italic. Deduced functional domains based on sequence homologies with related viruses are presented as follows: X1; X2; NTB, nucleoside triphosphate binding protein or putative helicase; VPg, viral genome-linked protein; Pro, protease; Pol, polymerase; HAM1, putative inosine triphosphate pyrophosphatase; MP, movement protein; CP1, CP2, CP3, capsid protein 1,2, and 3, respectively.

The genomic organization of the RNA2 polyprotein was consistent with that of other cheraviruses, with three predicted cleavage sites (Figure 7, Table 4 and Table 5), delineating a putative movement protein (MP) and three capsid subunits (CP1-CP3) [26,47,48,49]. These proposed cleavage sites included a D (or E) at the −1 position, a small amino acid (G, A, M, or S) at the +1 position, and a highly conserved G at the −2 position.

Previous analyses of the cheravirus P1 polyproteins had identified four cleavage sites, delineating five protein domains (Pro-Co, NTB, VPg, Pro, and Pol) [26,47,49,50]. We identified putative cleavage sites that aligned well with these four cleavage sites and were consistent with the consensus sequence derived from the analysis of the cassava Congo cheravirus P2 polyprotein described above. The sequence GD/A was predicted as a putative cleavage site upstream of the NTB domain. This cleavage site aligned well with cleavage sites previously reported for other cheraviruses (GQ/G for other cheraviruses). A tentative GD/T cleavage site is proposed at the VPg-Pro junction for the cassava Congo cheravirus isolates, corresponding to the putative GQ/G or GE/G cleavage sites identified for other cheraviruses. The proposed NTB-VPg cleavage site is GQ/G (Table 4). This cleavage site was identical to that of ALSV and CuLV and aligned well with cleavage sites predicted for other cheraviruses. Cleavage between the Pro and Pol domain could occur at one of the two possible cleavage sites suggested in Table 4 (AQ/A or LD/A). These two putative cleavage sites are in proximity to each other, separated by only two amino acids.

Further analysis identified an additional cleavage site in the N-terminal region of the polyprotein that would delineate two protein domains upstream of the NTB domain, similar to the X1 and X2 domains previously identified in the P1 polyprotein of viruses in the genera *Nepovirus* and *Sadwavirus* [19,37,38,40,51]. The proposed cassava Congo cheravirus X1-X2 cleavage site has a sequence of GD/G, which is consistent with the cleavage site consensus sequence emerging for this virus. We reexamined the P1 polyprotein sequence of other cheraviruses. We could also find putative X1-X2 cleavage sites at a corresponding position for all cheraviruses, suggesting that the presence of two protein domains upstream of the NTB domain is a conserved feature in the genus *Cheravirus*.

We also noted that the C-terminal region of the P1 polyprotein (downstream of the Pro-Pol cleavage site) is longer than that observed in other cheraviruses, with a possible additional protein domain downstream of the polymerase (the Ham1 domain, which encodes an ITPase; see below). We searched the polyprotein for an additional cleavage site. We identified a putative cleavage site downstream of the Pol domain that was generally consistent with the consensus for other cleavage sites in the P1 polyprotein (FMSAD/GL), with the G at the −2 position replaced by another small amino acid. Thus, our analysis identifies six cleavage sites in the RNA1-encoded polyprotein, delineating seven protein domains (Figure 7).

Most putative cleavage sites included an aspartic acid (D) at the −1 position, although two putative cleavage sites had a Q at the −1 position (NTB-VPg and possibly Pro-Pol cleavage sites). A glycine (G) was most often found in the +1 position of the cleavage sites, although some putative cleavage sites also had other small amino acids (T, A, S or M) at this position. Comparing the consensus cleavage site of the cassava Congo cheravirus isolates to those of other cheraviruses previously described, the strong preference for an aspartic acid (D) in the −1 position is unique to cassava Congo cheravirus isolates (Table 5). This preference has not yet been described for other viruses in the family *Secoviridae*. The protease of blackcurrant reversion virus, a nepovirus, prefers an N at the −1 position of the cleavage sites and occasionally recognizes cleavage sites with a D at the −1 position [40].

**Figure 8:**
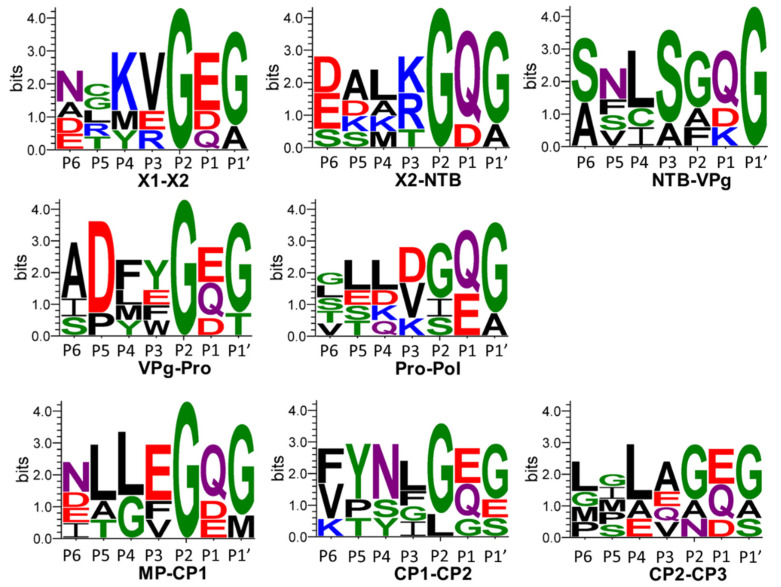
Sequence logos from the P6 to P1′ positions of cleavage sites corresponding to the cheravirus genus, including the CaSeCoV (the exemplar isolate CGO-BC), and a single isolate for each apple latent spherical virus (ALSV), cherry rasp leaf virus (CRLV), currant latent virus (CuLV), and arracacha virus B (AVB). The number of cleavage site sequences included in each sequence logo is N = 7. Sequence logos were generated as described in the methodology, using confidently predicted or experimentally validated cleavage sites listed in Table 4. Amino acid colours correspond to the chemistry: polar amino acids (G, S, T, Y, C, green), neutral amino acids (Q, N, purple), basic amino acids (K, R, H, blue), acidic amino acids (D, E, red) and hydrophobic amino acids (A, V, L, I, P, W, F, M, black).

In conclusion, the putative cleavage site consensus for the cassava Congo cheravirus is (G, a) (D, Q)/(G, A, t, s, m). The comparison of this consensus to what was observed previously for other cheraviruses suggests a strong preference for a G at −2 position for all cheraviruses, including the ones reported in this study. The strong preference for a D at the −1 position in the cassava Congo cheravirus is somewhat unique. However, the predicted NTB-VPg cleavage site for CRLV also has an aspartic acid (D) at the −1 position (Table 4). Considering findings from [26], who previously compared cleavage sites of three characterized cheraviruses, the putative cleavage-site consensus for viruses in the genus Cheravirus can be summarized as (G, a) (Q, E, D)/(G, s) (Figure 8).

### 3.7. Protein Domains in the N-Terminal Region of the RNA1 Polyprotein

The X1 protein is highly variable among nepoviruses and cheraviruses, and its function remains unknown [40]. Similarly, this domain was the most variable in size amongst the cassava Congo cheravirus isolates. The X1 domain of the isolate CGO-BC was the shortest (418 aa) while that of CGO-SK12 was the longest (531 aa).

**Figure 9:**
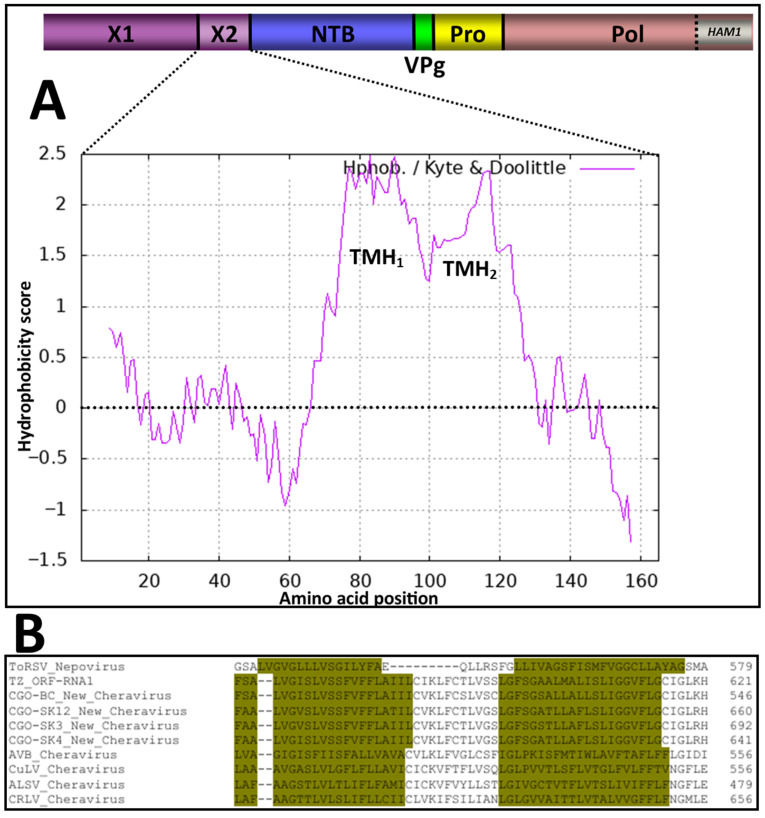
Computer-assisted prediction of transmembrane helices (TMH) in the X2-encoded domain of the RNA1 segment: (**A**) The hydrophobicity plot of X2, calculated using the algorithm of Kyte and Doolittle with a window size of 17 amino acids [53]. The RNA1-encoded polyprotein is shown at the top of the panel, with the indicated protein domains. Vertical lines represent the putative cleavage sites recognized by the RNA1-encoded 3C-like proteinase of the new cheravirus. (**B**) Multiple amino acid sequence alignment of the X2 domains for the new cheravirus isolates, along with other existing cheraviruses, showing amino acid residues associated with the putative double trans-membrane helices for a 2-pass transmembrane X2 domain of viruses reported in this study similar to that of the experimentally confirmed ToRSV (nepovirus) (dark-yellow highlighting in alignment). Amino acids are numbered from the first amino acid of the P1 polyprotein.

The function of the X2 domain has previously been investigated in the nepovirus tomato ringspot virus (ToRSV). The X2 protein crosses the membrane twice since it possesses two transmembrane helices [52]. Using the algorithm of Kyte and Doolittle with a window size of 17 amino acids [53], two hydrophobic domains (putative transmembrane helices) were also predicted in the X2 domain of the cassava isolates, as well as that of other cheraviruses (Figure 9).

With sizes varying between 906 and 916 amino acids for the six isolates, the polymerase was the longest protein of the RNA1-encoded polyprotein. The “Pro-Pol” sequence, defined as the sequence between the conserved catalytic cysteine (or serine) of the 3C-like protease (CG) and the GDD motif of the RNA-dependent RNA polymerase, characteristic of viruses in the family *Secoviridae* (used for phylogenetic classification) [19], was quite “particular” for the viruses described in this study. They had an insertion of 36 additional amino acids, an uncommon feature compared to the sequences of all other included viruses (Figure S3).

### 3.8. A Putative Maf/HAM1 Motif Within the Polymerase Domain

**Figure 10:**
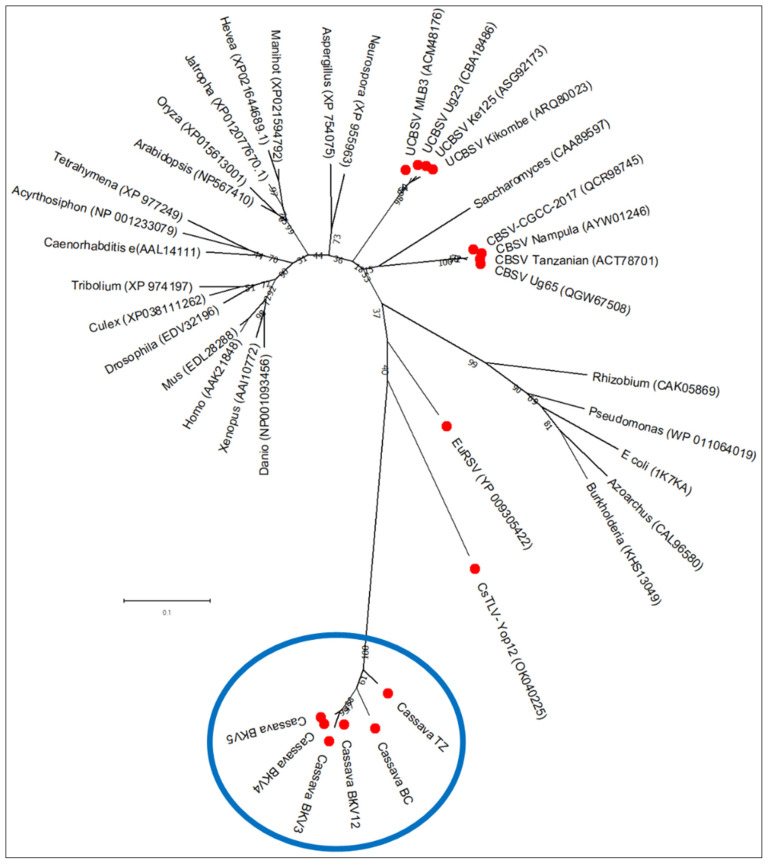
Phylogenetic relationships of Maf/HAM1 motifs between viruses reported in this study (framed in blue) with those of representative members of different kingdoms of life (viruses are pointed in solid red circles). The alignment was generated using ClustalW integrated into MEGA X, and a maximum-likelihood phylogenetic tree was reconstructed in MEGA X using the LG + G model for amino acid sequence alignments. Detailed information on these sequences (complete names) is provided in Table S6.

The RNA1-encoded polymerase displayed an insertion of 36 amino acids and contained an extended C-terminal region with similarity to inosine triphosphate pyrophosphatases (ITPases; HAM1 family). This putative HAM1 domain was approximately 232 amino acids in length and likely cleaved from the polymerase at a consensus site FMSAD/GL) (Figure S5). Phylogenetic analysis grouped the viral HAM1-like proteins with those from cassava torrado-like virus (CsTLV) and euphorbia ringspot virus (EuRSV), forming a clade adjacent to bacterial ITPases (Figure 10).

The exact function of the viral ITPase has not been broadly characterized except for investigations by [30], who proposed that the presence of Ham1 proteins with highly conserved ITPase motifs for the U/CBSV served as a necrosis determinant in *Nicotiana benthamiana* and could act as a selection determinant during infections of cassava. Moreover, ref. [54] categorized key residues/motifs that influence ITPase substrate specificity in human ITPase. They identified three essential, four intermediates, and one dispensable amino acid residue.

To investigate whether crucial residues from the human ITPase active sites are conserved in ITPases from other organisms, including the viruses reported in this study, we performed an amino acid alignment (Figure S6). The results of this alignment are summarized in Table 6.

Alignment of active-site residues revealed conservation of two essential amino acids (arginine and tryptophan) and conservative substitutions at the third (glutamic acid to aspartic acid). This pattern parallels findings in CsTLV and contrasts with *Potyviridae* HAM1 proteins (e.g., CBSV, UCBSV), where all key residues are conserved [30,54].

These observations suggest that the viral HAM1 motif identified for cassava Congo cheraviruses could be functionally conserved, potentially influencing substrate specificity and pathogenicity during cassava infections.

## 4. Discussion

This study reports the genome characterization of cassava Congo cheravirus, the first cheravirus documented in cassava. Genome architecture, including a bipartite organization and the presence of three coat proteins, is consistent with viruses of the genus *Cheravirus.* However, two new features were identified for the P1 polyprotein: (i) the presence of two domains (X1 and X2) upstream the NTB region, which was previously only identified in members of the genera *Nepovirus* (Sanfacon, 2022) [37,38], and *Sadwavirus* [51], and appears to be conserved in all characterized members of the genus *Cheravirus*; and (ii) a unique Maf/HAM1-like inosine triphosphatase (ITPase) motif, not present in other cheraviruses and only previously identified in a few viruses infecting euphorbiaceous hosts [15,24,25,55,56]. The latter may confer a selective advantage in cassava by mitigating the incorporation of non-canonical nucleotides, a hypothesis that requires experimental validation [56].

A notable outcome of this study is the detailed prediction of P1 and P2 cleavage sites based on multiple sequence alignment with well-characterized secovirids. While experimental validation is pending, the congruence between alignment-based predictions and SDS-PAGE evidence for the three coat proteins provides high confidence in the proposed proteolytic map. The identification of two cleavage sites upstream of the NTB further strongly supports the presence of X1 and X2 domains, a newly documented feature for this genus [51]. Additionally, the putative consensus cleavage peptide motifs exhibit a distinct preference for aspartic acid at the −1 position, suggesting functional divergence in protease specificity relative to other cheravirus members. This refined proteolytic framework offers a valuable basis for future mutagenesis and functional studies [57].

Phylogenetic analysis placed the virus within the genus Cheravirus, with amino acid identities in both the conserved Pro-Pol and coat protein regions falling well below the ICTV species demarcation thresholds. Isolates from Bas-Congo, South-Kivu, and Tanzania formed three distinct clusters, and recombination signals in both RNA segments suggest ongoing genetic exchange. The recovery of viral sequences from historical RNAseq datasets indicates that the virus has circulated undetected for years, potentially aided by mild or latent symptom expression.

From a taxonomic perspective, the virus meets species-level criteria for *Cheravirus* but does not satisfy genus-level demarcation criteria to warrant reclassification [58]. The identification of the HAM1 motif within a *Cheravirus* genome expands the functional diversity known within this genus and may inform studies of viral adaptation in perennial and woody euphorbiaceous hosts [27].

The biological characterization of the newly identified virus has achieved several steps proposed in a recently published framework [59]. Viral particles of this proposed new species have been observed, the expression of the coat protein validated, and its mechanical transmission to *N. benthamiana* demonstrated. In addition, we report its presence in two countries, at locations at least 1700 km apart. A more in-depth study in South Kivu province underscored its widespread distribution (32% field prevalence and an average of 40% individual-plant incidence per field). This relatively high prevalence might suggest a common asymptomatic presence in propagated planting material, or rapid horizontal dissemination within or between fields, or from other ecosystems.

The geographical distribution of infected fields indicates heterogeneity, with the virus concentrated in specific foci rather than evenly spread across the Uvira territory. The coexistence of high-prevalence fields in proximity with lower-prevalence/negative fields could indicate recent introduction followed by local spread. Alternatively, this situation could reflect the persistence of vegetative propagation practices. Whatever the situation, this coexistence suggests that local transmission is incomplete, and virus spread may depend heavily on movement of cutting between farms rather than airborne or insect-only transmission.

Cheraviruses are classically transmitted by soil-borne nematodes, whose limited mobility typically results in aggregated or patchy distributions of infected plants within fields. The spatial distribution observed for cassava Congo cheravirus, characterized by localized hotspots interspaced with virus-free fields, is consistent with such transmission dynamics. However, cassava is predominantly propagated vegetatively, and the exchange of infected cuttings between neighboring farms [32] may also contribute to clustered infection patterns. In the absence of direct evidence for nematode vectors in this study, the observed distribution should therefore be interpreted as compatible with, but not diagnostic of, nematode-mediated transmission. Identification of potential vectors and the development of transmission assays will be required to clarify the epidemiological drivers of viral spread.

Additionally, the geographical concentration of highly prevalent fields in the Western part of the Uvira Territory highlighted that this virus is more prevalent in areas with more widespread cassava cultivation [32], located close to Burundi and Rwanda, which might also facilitate cross-border dissemination. This situation would raise concern about potential regional spread via the exchange of planting material, provided that it has a significant impact on yield.

Future research opportunities include characterizing potential transmission routes and vectors, and expanding symptomology and yield impact assessments. Performing functional assays for ITPase would also bring more insights into this function identified for virus infecting *Euphorbiaceae* hosts, including two recently discovered viruses carrying this motif [27].

In conclusion, all cassava isolates described in this study represent a single novel species within the genus *Cheravirus*. This virus, the “cassava Congo cheravirus”, has unique genomic traits that may influence its interactions with cassava and its epidemiology. Its detection in geographically distant regions underscores the importance of coordinated surveillance and functional studies to determine its roles in cassava growth and health.

## Figures and Tables

**Table 1:** Genome organization of the 13 contigs reconstructed in this study.

RNA1							
Contig Names	Genome Length	5′UTR	ORF1		3′UTR	Poly-A Tail	Accession Number (Genbank)
	nt	nt	nt	aa	nt	nt	
CGO-BC (reference)	7380	131	7110	2369	139	Yes	ON_924323
CGO-SK3	7702	171 * ^(6)^	7380	2460	153	Yes	OP440440
CGO-SK4	7581	37 * ^(144)^	7391	2463	153	Yes	OP440437
CGO-SK5	7708	166 * ^(10)^	7389	2462	153	Yes	OP440438
CGO-SK12	7766	163 * ^(10)^	7449	2482	154	Yes	OP440439
TZ	7638	171 * ^(5)^	7347	2449	120	No	OP440441
RNA2							
Contig names	Genome length	5′UTR	ORF1		3′UTR	Poly-A tail	Accession number (GenBank)
	nt	nt	nt	aa	nt	nt	
CGO-BC (reference)	3444	95	3210	1069	139	Yes	ON_924324
CGO-SK3	3449	110 * ^(8)^	3204	1067	150	Yes	OP440433
CGO-SK4	3457	108 * ^(10)^	3204	1067	150	Yes	OP440432
CGO-SK5	3445	109 * ^(9)^	3204	1067	152	Yes	OP440431
CGO-SK12	3430	103 * ^(8)^	3204	1067	151	Yes	OP440434
TZ-55	3457	109 *	3195	1065	153	No	OP440436
TZ-56	3449	96 * ^(6)^	3195	1065	158	No	OP440435

*: The 5′ ends of these contigs were not fully reconstructed. The number of missing nucleotides for each genome compared to the fully reconstructed one is shown in brackets.

**Table 2:** Range of identity percentages for the conserved “Pro-Pol” region and the coat protein amino acid sequences between isolates described in this study and viruses included in representative species of various genera in the family *Secoviridae*.

Genus	Representative Viruses	Pro-Pol		CP	
		Min	Max	Min	Max
*Cheravirus*	Cherry rasp leaf virus	45%	46%	8%	9%
*Torradovirus*	Tomato torrado virus	37%	38%	10%	11%
*Waikavirus*	Rice tungro spherical virus	32%	33%	11%	12%
*Sequivirus*	Parsnip yellow fleck virus	37%	37%	10%	11%
*Sadwavirus*	Satsuma dwarf virus	30%	30%	10%	11%
*Nepovirus*	Tomato ringspot virus	30%	32%	8%	10%
	Grapevine fanleaf virus	34%	34%	10%	10%
	Beet ringspot virus	32%	33%	8%	10%
*Comovirus*	Cowpea mosaic virus	33%	34%	8%	10%
*Fabavirus*	Broadbean wilt virus 2	35%	36%	7%	8%

**Table 3:** Intergroup and intragroup average pairwise identity and standard deviation were calculated for the “Pro-Pol” region, the CP block, the RNA1 and RNA2-encoded polyproteins (P1 and P2, respectively), and the nucleotide sequences of (nearly) complete genomes of the RNA1 and RNA2 segments.

		Within the Kivu Isolates	Within Tanzania Isolates	Between Kivu & Tanzania Isolates	Between Kivu & Bas-Congo Isolates	Between Bas-Congo & Tanzania Isolates
Pro-Pol	aa identity	97% ± 0.5%	-	86% ± 1.5%	87% ± 1.5%	84% ± 1.7%
CP block	aa identity	98% ± 0.4%	97% ± 0.8%	70% ± 2%	81% ± 2%	75% ± 2%
P1 polyprotein	aa identity	94% ± 0.4%	-	79% ± 0.8%	82% ± 0.8%	80% ± 0.8%
P2 polyprotein	aa identity	97% ± 0.4%	95% ± 0.6%	74% ± 1%	71% ± 1%	73% ± 1%
RNA1 genome	nt identity	90% ± 0.2%	-	75% ± 0.5%	75% ± 0.5%	75% ± 0.5%
RNA2 genome	nt identity	95% ± 0.3%	89% ± 0.6%	72% ± 0.7%	70% ± 0.8%	73% ± 0.8%

**Table 4:** Location of putative cleavage sites predicted in the RNA1 and RNA2-encoded polyproteins for isolates identified in cassava plants in this study.

RNA1 Polyprotein						
Domains	CGO-SK3	CGO-SK4	CGO-SK12	CGO-BC	TZ	Consensus
X1-X2	GVGD ^565^/GL	VGVGD ^514^/GL	VGVGD ^533^/GM	MVGD ^419^/GF	MVGD ^493^/GF	GD/G
X2-NTB	MKGD ^730^/AI	MKGD ^681^/AI	MRGD ^698^/AI	MKGD ^585^/AI	MOGD ^657^/AI	GD/AI
NTB-VPg	ISGQ ^1310^/GD	ISGQ ^1261^/GD	ISGQ ^1314^/GD	ISGQ ^1165^/GD	ISGQ ^1237^/GD	GQ/GD
VPg-Pro	LYGD ^1365^/TQ	LYGD ^1316^/TQ	LYGD ^1369^/TQ	MYGD ^1220^/TQ	MYGD ^1292^/TQ	GD/TQ
Pro-Pol	LDAQ ^1609^/AR	LDAQ ^1560^/AR	LDAQ ^1613^/AR	LDSQ ^1463^/AR	LDAQ ^1535^/AQ	AQ/AR
	VLLD ^1607^/AQ	VLLD ^1558^/AQ	VLLD ^1611^/AQ	VLLD ^1461^/SQ	VLLD ^1533^/AQ	LD/AQ
Pol-Ham1	FMSAD ^2283^/GL	FMSAD ^2233^/GL	FMSAD ^2286^/GL	FMSAD ^2137^/GL	FMSAD ^2210^/GL	AD/GL
**RNA2 Polyprotein**						
Domains	CGO-SK3	CGO-SK4	CGO-SK12	CGO-BC	TZ	Consensus
MP-CP1	GTGD ^447^/ML	GTGD ^447^/ML	GTGD ^456^/ML	GFGD ^447^/ML	AKGD ^447^/ME	GD/ML
CP1-CP2	SFGD ^623^/GG	SFGD ^623^/GG	SFGD ^632^/GG	SFGE ^624^/GG	SFGD ^618^/GG	GD/GG
CP2-CP3	PSGD ^868^/SL	PSGD ^868^/SL	PSGD ^877^/SL	AQGD ^869^/AL	AQGD ^863^/AL	GD/SL

Numbering corresponds to the amino acid position starting from the beginning of the polyprotein. The red highlight is filled when this consensus is met by isolates described in this study at the −1 position. The grey highlight for both proposed Pro-Pol cleavage sites indicates the two potential cleavage sites at the Pro-Pol junction.

**Table 5:** Consensus cleavage sites of isolates from this study and those of previously described viruses from the genus *Cheravirus.*

RNA1-Polyprotein					
Sites	New cheravirus	ALSV	CRLV	CuLV	AVB
X1-X2	GD/GL	NTKEGQ/GP	NCKVGE/GP	EGKRGE/GP	DRYVGE/AG
X2-NTB	GD/AI	EALRGQ/GL	DDLRGQ/GV	DAKKGQ/GI	SSATGQ/GP
NTB-VPg	GQ/GD	SSLSAQ/GP	SNLSGD/GA	SNLSGQ/GP	AFCAFK/GE
VPg-Pro	GD/TQ	IPLWGQ/GP	ADFFGE/GP	SDYEGQ/GP	ADFYGE/GP
Pro-Pol	AQ/AR	SEKVGQ/GP	LSDKGQ/GP	GTLVGE/GP	TLQDIE/GA
	LD/AQ				DFCAGE/VA
Pol-Ham1	AD/GL	-	-	-	-
**RNA2 Polyprotein**					
Sites	This study	ALSV	CRLV	CuLV	AVB
MP-CP1	GD/ML	NLLEGQ/GP	NLLEGQ/GP	NLLEGQ/GP	IAGVGE/GP
CP1-CP2	GD/GG	FYNIGQ/GA	VYNLGQ/GQ	FYNLGE/SN	SEYHGN/AT
CP2-CP3	GD/SL	GPLVGE/GS	PILAAE/GP	LSLEGQ/GP	GFSLGE/AN

The red and green highlights are filled when consensus is met at the −1 and +1 positions of the cleavage site, respectively. The grey highlight indicates the two potential consensus cleavage sites at the Pro-Pol junction.

**Table 6:** Comparison of conserved amino acid residues in ITPases of representative members of various kingdoms of life (including viruses reported in this study) to essential amino acid residues that influence substrate specificity in human ITPase.

N°	Essential Amino Acid Residues in Human ITPase	Correspondent Amino Acid Residues in Alignment with:					
		*E. coli*	*S. cerevisiae*	CsTLV	EuRSV	CBSV & UCBSV	New Cheravirus
		Essentials					
1	Glutamic acid (E_22)	E	E	E	E	E	D
2	Tryptophan (W_151)	Y	W	F	W	W	W
3	Arginine (R_178)	R	R	R	R	R	R
		Intermediates					
4	Phenylalanine (F_149)	F	F	F	F	F	Y (tyrosine)
5	Aspartic acid (D_152)	D	D	D	D	D	E
6	Lysine (K_152)	K	K	K	K	K	S
7	Serine (S_176)	S	S	G (glycine)	S	S	C (cysteine)
		Dispensable					
8	Histidine (H_177)	H	H	L (leucine)	H	H	A (alanine)

Table cells are filled green when the residue is conserved relative to the human ITPase; otherwise, the cells are filled orange.

## Data Availability

The sequencing reads generated through this study are available via the Sequence Reads Archive as a bioproject reference PRJNA1311516 (SRR35169868, SRR35169867, SRR35169866, SRR35169865). The genome sequence datasets generated and analyzed in this study are available in the GenBank repository under the following accession numbers: ON_924323, OP440440, OP440437, OP440438, OP440439, OP440441, ON_924324, OP440433, OP440432, OP440431, OP440434, OP440436, OP440435.

## Supplementary Materials

The following supporting information can be downloaded at: https://www.mdpi.com/article/10.3390/v18010084/s1, Table S1: Lengths, number of reads integrated, percent of total reads, and average coverage for viruses included in the family *Secoviridae* contigs from the various analyzed pools of cassava samples. Table S2: Complete names of selected *Secoviridae* members used for the phylogenetic study. Table S3: Primers used for Confirmatory RT-PCR, RACE, and Sanger sequencing for HAM1 motif confirmation. Table S4: Accession numbers and abbreviations for selected *Secoviridae* members used to deduce cleavage sites and functional domains of the RNA1 and RNA2 encoded polyproteins (respectively P1 and P2). Figure S1: Multiple amino acid sequence alignment of the protease domain localises the conserved Histidine of the substrate binding pocket region and the protease catalytic Cysteine. Figure S2: Phylogenetic analysis of the coat protein amino acid for the five isolates detected in cassava (strong green triangles and green frame) and of type isolates from recognized species in the family *Secoviridae.* Figure S3: Multiple amino acid sequence alignment of the conserved “CG-GDD” motif shows the insertion of 36 amino acids (highlighted in red) upstream of the “GDD” motif in the polymerase domain. Figure S4: Phylogenetic trees reconstructed using (**A**) the amino acid sequences of the “Pro-Pol” region, (**B**) the amino acid sequences of the entire P1 polyprotein, (**C**) the complete/nearly nucleotide sequence of the RNA1 segment (**D**) the amino acid sequences of the coat protein block, (**E**) the amino acid sequences of the entire P2 polyprotein and (**F**) the nearly/complete nucleotide sequence of the RNA2 segment for the new *Secoviridae* members described in this study. Figure S5: Multiple amino acid sequence alignment of the C-terminal region of the polymerase domain of viruses reported in this study, together with the arracacha virus A. The figure shows a putative cleavage site (FMSAD/G) that could delineate a putative domain downstream of the polymerase. Table S5: Recombination events detected among isolates from the new cheravirus. Figure S6: Multiple amino acid sequence alignment showing conserved sites of ITPases from representative members of various kingdoms of life. The alignment was built using Mview [60]. Table S6: Complete names and accession numbers for representative members of various kingdoms of life used for phylogenetic study of the HAM1 motifs.
