# Supplementary material for: Genomic Organization of the Newly Discovered Cassava Congo Cheravirus Reveals a Unique Maf/HAM1 Motif in the C-Terminal Region of the RNA1 Polyprotein and Suggests the Presence of Two Protein Domains Upstream of the Putative Helicase Domain
Source: Viruses. 2026 Jan 8;18(1):84. doi: 10.3390/v18010084 (PMC12846526; doi:10.3390/v18010084)

I. Supplementary material

Table S1. Lengths, number of reads integrated, percent of total reads, and average coverage for the *Secoviridae* contigs from the various analyzed pools of cassava samples.

Origin of samples		Isolate name	Fields /pool	Positive fields/pool	Positive individual plants/field[a]	Genome segment	Contig name	Contig length [nt]	Mapped reads	Average genome coverage	SRA accession	Biosample accession
Country	Province											
**Congo DR**	South-Kivu	CGO-SK3	7	5	27	RNA1[b]	CGO-SK3-RNA1	7,71'0	57,769	1,064.6	SRR35169868	SAMN50810141
						RNA2[b]	CGO-SK3-RNA2	3,502	18,723	765.6		
		CGO-SK4	8	7	33	RNA1[b]	CGO-SK4-RNA1	7,61'0	54,876	1,038.9	SRR35169867	SAMN50810142
						RNA2[b]	CGO-SK4-RNA2	2,871	37,045	1,886.4		
		CGO-SK5	7	5	31	RNA1[b]	CGO-SK5-RNA1	7,718	64,395	1,098.8	SRR35169866	SAMN50810143
						RNA2[b]	CGO-SK5-RNA2	3,681	18,121	705.9		
		CGO-SK12	11	6	39	RNA1[b]	CGO-SK12-RNA1	7,793	58,827	1,236.6	SRR35169865	SAMN50810144
						RNA2[b]	CGO-SK12-RNA2	3,477	18,545	882.7		
	Bas-Congo	CGO-BC	-	-	-	RNA1[c]	CGO-BC-RNA1	7,405	-	-	-	-
						RNA2[c]	CGO-BC-RNA2	3,46'0	-	-		
**Tanzania**		TZ	-	-	-	RNA1[d]	TZ-RNA1	7,637	179,438	3,007	-	SAMN50810145
						RNA2_55[d]	TZ-RNA2_55	3,533	59,753	1,869		
						RNA2_56[d]	TZRNA2_56	3,477	86,555	2,548		

a = Not all individual plants could be included in the RT-PCR test as the stock of dried leaf samples ran out and could no longer be retrieved through the experimental field (some stem cuttings failed to grow in the experimental field, while others died during the experiment). **B** = only 3' extremity could be completed by RACE experiments. **C** = both 3' and 5' extremities were completed through RACE. **D** = not included in the RACE experiment. Contigs were named considering the initial letters of the code of the country where the samples were collected-initial letters of the province of origin (when available)-and a number indicating the reference of the HTS pool (when available).

Table S2. Complete names of selected *Secoviridae* members used for the phylogenetic study.

Genus	Species names	Abbreviation	Accession numbers	
			RNA1 (Pro-pol)	*RNA2 (CP)*
Cheravirus	Arracacha virus B	AVB	JQ437415	JQ581051
Cheravirus	Apple latent spherical virus	ALSV	AB030940	AB030941
Cheravirus	Cherry rasp leaf virus	CRLV	AJ621357	AJ621358
Cheravirus	Currant latent virus	CuLV	KT692952	KT692953
Cheravirus	Stocky prune virus	StPV	DQ143874	DQ143875
Comovirus	Bean pod mottle virus	BPMV	M62738	U70866
Comovirus	Cowpea mosaic virus	CPMV	X00206	X00729
Comovirus	Cowpea severe mosaic virus	CPSMV	M83830	M83309
Comovirus	Radish mosaic virus	RaMV	AB295643	-
Comovirus	Red clover mottle virus	RCMV	X64886	M14913
Comovirus	squash mosaic virus	SqMV	AB054688	AB054689
Comovirus	Broad bean true mosaic virus	BBTMV	GU810903	GU810904
Comovirus	bean rugose mosaic virus	BRMV	-	KP404603
Comovirus	Andean potato mottle virus	APMV	-	L16239
Fabavirus	Broad bean wilt virus 1	BBWV1	AB084450	AB084451
Fabavirus	Broad bean wilt virus 2	BBWV2	AF225953	AF225954
Fabavirus	Cucurbit mild mosaic virus	CuMMV	EU881936	EU881937
Fabavirus	Grapevine fabavirus	GFabV	KX241482	KX241485
Fabavirus	Prunus virus F	PrVF	KX269865	KX269871
Fabavirus	lamium mild mosaic virus	LMMV	KC595304	KC595305
Fabavirus	Gentian mosaic virus	GeMV	AB084452	AB084453
Nepovirus	Aeonium ringspot virus	AeRSV	JX304792	JQ670669
Nepovirus	Arabis mosaic virus	ArMV	AY303786	AY017339
Nepovirus	Artichoke yellow ringspot virus	AYRSV	AM087671	-
Nepovirus	blueberry latent spherical virus	BLSV	AB649296	AB649297
Nepovirus	Beet ringspot virus	BRSV	D00322	X04062
Nepovirus	blackcurrant reversion virus	BRV	AF368272	AF020051
Nepovirus	cherry leaf roll virus	CLRV	FR851461	FR851462
Nepovirus	cycas necrotic stunt virus	CNSV	AB073147	AB073148
Nepovirus	Grapevine Bulgarian latent virus	GBLV	FN691934	FN691935
Nepovirus	Grapevine chrome mosaic virus	GCMV	X15346	X15163
Nepovirus	Grapevine fanleaf virus	GFLV	D00915	X16907
Nepovirus	Melon mild mottle virus	MMMoV	AB518485	AB518486
Nepovirus	Mulberry mosaic leaf roll-associated virus	MMLRaV	KC904083	KC904084

Genus	Species names	Abbreviation	Accession numbers	
			RNA1 (Pro-pol)	*RNA2 (CP)*
Nepovirus	Peach rosette mosaic virus	PRMV	KY646466	KJ572573
Nepovirus	Raspberry ringspot virus	RpRSV	AY303787	AY303788
Nepovirus	tomato black ring virus	TBRV	AY157993	AY157994
Nepovirus	Tomato ringspot virus	ToRSV	L19655	D12477
Nepovirus	Potato black ringspot nepovirus	PBRSV	KC832887	KC832892
Nepovirus	Potato virus B	PVB	KX656670	KX656671
Nepovirus	Grapevine anatolian ringspot virus	GARSV	HE774604	AY291207
Nepovirus	Artichoke Italian latent virus	AILV	LT608395	LT608396
Nepovirus	Soybean latent spherical virus	SLSV	KX424571	KX424572
Nepovirus	Grapevine deformation virus	GDefV	HE613269	AY291208
Nepovirus	Tobacco ringspot virus	TRSV	U50869	AY363727
Nepovirus	Olive latent ringspot virus	OLRSV	-	AJ277435
Sadwavirus	Black raspberry necrosis virus	BRNV	DQ344639	DQ344640
Sadwavirus	Chocolate lily virus A	CLVA	JN052073	JN052074
Sadwavirus	Dioscorea mosaic-associated virus	DMaV	KU215538	KU215539
Sadwavirus	satsuma dwarf virus	SDV	AB009958	AB009959
Sadwavirus	Strawberry mottle virus	SMoV	AJ311875	AJ311876
Sequivirus	Carrot necrotic dieback virus	CNDV	EU980442	EU980442
Sequivirus	Parsnip yellow fleck virus	PYFV	D14066	D14066
Torradovirus	Carrot torradovirus 1	CaTV1	KF533719	KF533720
Torradovirus	Lettuce necrotic leaf curl virus	LNLCV	KC855266	KC855267
Torradovirus	Motherwort yellow mottle virus	MYMoV	KM229700	KM229701
Torradovirus	squash chlorotic leaf spot virus	SCLSV	KU052530	KU052531
Torradovirus	Tomato marchitez virus	ToMarV	EF681764	EF681765
Torradovirus	tomato torrado virus	ToTV	DQ388879	DQ388880
Stralarivirus	Strawberry latent ringspot virus	SLRSV	AY860978	AY860979
Waikavirus	bellflower vein chlorosis virus	BVCV	KT238881	KT238881
Waikavirus	maize chlorotic dwarf virus	MCDV	U67839	U67839
Waikavirus	Rice tungro spherical virus	RTSV	M95497	M95497
Enterovirus	Enterovirus C	EVC	NP_041277	

Table S3. Primers used for Confirmatory RT-PCR, RACE and Sanger sequencing for HAM1 motif confirmation.

Target genome segment	Segment ends	lab manipulation	Primer Name	Sequence 5'-3'	Length	Annealing T°	Product
**RNA1**	3' end	RACE	5658F	TGCGCTCCTTGCTGATGATGGCCTCA	26nt		
			546F	ATAAGGCGGCAGGGAAGGTGGGTATT	26nt		
		Confirmatory RT-PCR	7539R3	GCGGATGCGATTATTGACTTTCA	23nt	60,2	813bp
			8456F	CTGATGGATTGTCTCAGGCCATTC	24nt		
	5' End	RACE	2947R	AGCCTCTCGATCAGCAGCCTCAGTGT	26nt		
			4722R	AGGCAATCACCTCGGGCTTGAGCATC	26nt		
		Confirmatory RT-PCR	129F5	TCTGCAAAGATCCTTTCCTCTGGT	24nt	61,7	454bp
			583R5	CCGGCAGTAACATTAGCAGCAAA	23nt		
**RNA2**	3' end	RACE	815F	GGGCCAAGGCTCACCAGTCAAATGCA	26nt		
			855F	GACCATCGGTGGGAAGGTGACTGGAGA	27nt		
		Confirmatory RT-PCR	2388F	GCGGAGGAAGAGGGTATTGTTAT	23nt	60,9	396bp
			2784R	CCAACCACATCCTATCATCCCAT	23nt		
	5' End	RACE	2176R	GGAGCACGCCATTGGGAGTCCAGTT	25nt		
			1680R	TCTGATAGGCACTACCAGCATCACTTGA	28nt		
		Confirmatory RT-PCR	34F	CTTCTGCTCTCTGCTCTTGATCA	23nt	60,5	576bp
			610R	GTATCCAGAAACCTCCCAGTGAA	23nt		
**HAM1 motif confirmation**			8456F	CTGATGGATTGTCTCAGGCCATTC	24nt	65,2	664bp
			9120R	ATGCGGACTTGTTCTCCACAGTGA	24nt		

Table S4. Accession numbers and abbreviations for selected *Secoviridae* members used to deduce cleavage sites and functional domains of the RNA1 and RNA2 encoded polyproteins (respectively P1 and P2)

Species names	Genus	Abbreviation	Sequence accession numbers (GenBank)	
			RNA1	RNA2
Apple latent spherical virus	Cheravirus	ALSV	NC_003787.1	NC_003788.1
Cherry rasp leaf virus	Cheravirus	CRLV	NC_006271.1	NC_006272.1
Currant latent virus	Cheravirus	CuLV	NC_029038.1	NC_029036.1
Arracacha virus B	Cheravirus	AVB	NC_020898.1	NC_020897.1
Stocky prune virus	Cheravirus	StPV	NC_043388.1	NC_043387.1
Cowpea mosaic virus	Comovirus	CPMV	NC_003549.1	-
Arabis mosaic virus	Nepovirus	ArMV	NC_006057.1	-
Tomato ringspot virus	Nepovirus	ToRSV	NC_003840.1	-
Strawberry mottle virus	Sadwavirus	SMoV	NC_003445.1	-
Cassava Torrado-like virus	Torradovirus	CsTMV	MF449522.1	-
Black raspberry necrosis virus	Sadwavirus	BRNV	FN908128	FN908129
Chocolate lily virus A	Sadwavirus	CLVA	NC_016443	NC_016444
Dioscorea mosaic-associated virus	Sadwavirus	DMaV	KU215538	KU215539

Table S5. Recombination events detected among isolates from the new cheravirus

Recombination events	Recombinant isolate	Major parent	Minor parent	Nucleotide breakpoints (beginning-ending) of region derived from the minor parent	Average P-Value
**1**	CGO-SK3	CGO-BC	TZ-RNA1	226-406	5,094x10-21
**2**	CGO-SK5	TZ-RNA1	CGO-BC	189-403	1,936x10-02
**3**	CGO-SK5	CGO-SK12	CGO-BC	2062-2188	2,617x10-02
**4**	TZ-RNA1	CGO-SK3	CGO-SK4	1408-1547	3,176x10-02
**1**	CGO-SK12	CGO-SK5	CGO-SK3	1-88	6,249x10-10
**2**	TZ-55	CGO-BC	CGO-SK5	32-328	7,484x10-9
**3**	CGO-SK5	CGO-SK3	TZ-55	1313-1492	5,602x10-04
**4**	TZ-56	CGO-SK4	TZ-55	488-676	2,021X10-02
**5**	CGO-SK4	TZ-56	TZ-55	2922-3068	1,031X10-02
**6**	CGO-SK3	CGO-SK4	CGO-BC	2705-2860	3,204X10-02

Table S6. Complete names and accession numbers for representative members of various kingdoms of life used for phylogenetic study of the HAM1 motifs

Kingdoms	Complete names and abbreviations (when available) of organisms	Accession number
**Viruses**	Euphorbia ringspot virus (EuRSV-PV-0902)	YP_009305422
	Cassava brown streak virus- isolate Nampula (CBSV-Nampula)	AYW01246
	Cassava brown streak virus-isolate Tan_Z (CBSV-Tan_Z)	ACT78701
	Cassava brown streak virus-isolate Ug65 (CBSV-Ug65)	QGW67508

	Cassava brown streak virus-isolate CGCC-2017 (CBSV-CGCC-2017)	QCR98745
	Ugandan cassava brown streak virus-isolate kikombe (UCBSV-Kikombe)	ARQ80023
	Ugandan cassava brown streak virus-isolate MLB3 (UCBSV-MLB3)	ACM48176
	Ugandan cassava brown streak virus-isolate Ug_23 (UCBSV-Ug_23)	CBA18486
	Ugandan cassava brown streak virus-isolate Ke_125 (UCBSV-Ke_125)	ASG92173
	Cassava Torrado-like virus-Isolate Yop12 (CsTLV- Yop12)	OK040225
	Escherichia coli	1K7K_A
	Pseudomonas	WP_011064019
**Bacteria**	*Burkholderia multivorans*	KHS13049
	Azoarchus olearius	CAL96580
	Rhizobium leguminosarum bv. Viciae 3841	CAK05869
	Saccharomyces cerevisiae	CAA89597
**Fungi**	*Aspergillus fumigatus* Af293	XP_754075
	Neurospora crassa OR74A	XP_955963
	Arabidopsis thaliana	NP_567410
	Oryza sativa japonica group	XP_015613001
**Plantae**	*Manihot esculenta*	XP_021594792
	Jatropha curcas	XP_012077670.1
	Hevea brasiliensis	XP_021644689.1
	Caenorhabditis elegans	AAL14111
	Acyrthosiphon pisum	NP_001233079
	Drosophila ananassae	EDV32196
	Culex quinquefasciatus	XP_038111262
**Animalia**	*Tribolium castaneum*	XP_974197
	Xenopus laevis	AAI10772
	Danio rerio	NP_001093456
	Mus musculus	EDL28288
	Homo sapiens	AAK21848
	Tetrahymena thermophila SB210	XP_977249

Figure S1. Multiple amino acid sequence alignment of the protease domain localises the conserved Histidine of the substrate binding pocket region and the protease catalytic Cysteine.

```
                                         Protease catalytic Cys      Protease substrate-binding pocket
ToRSV_subgroup_C_nepovirus      ---NYSEGGDYSNDLPTSIISEYVNSPEDCGALLVAHLEG-----------GYKIIGMHV         1452
ArMV_subgroup_A_nepovirus       ---DEGGSAVYQNKIRRYIIYAHEAKRNDCGAIAVAEIQR-----------TPKVLAMLV         1438
AVA_Subgroup_A_Nepovirus        ---RTNGGYTYERILNKFIRVDGLAQDDDCGTLVATLIGG-----------QPRIVGMLV         1470
CsTLV_Torradovirus              GPIKYEGADGFIFSSTHSLRIRHTGMNGECGSVLFAPNLE---------NKQPVIVGIQC         1083
SMoV_sadwavirus                 -------KGRVVWQANNLLAAPLYHQVGHCGRLLLARDEA----------KCLKIVGIHV         1192
CPMV_comovirus                  ---------NYVNKVSRYLEYEAPTIPEDCGSLVIAHIGG-----------KHKIVGVHV         1132
AVB_Cheravirus                  ---------GL-LPLKKAYKYEMVTSPGFCGDVLLQVCSS-----------GVKILGMHT         1424
CuLV_Cheravirus                 ---------GS-LRMPPCYSYTFDTYPGLCTSPLICMSG-------G----RCILLGLHV         1424
ALSV_Cheravirus                 ---------GS-KPMPACYSYTFDTFAGLCTSPLISMDG-------G----RCVLLGLHV         1341
CRLV_Cheravirus                 ---------GS-KQMPACYSYVFETYAGLCTSPLIAQEG-------G----RCIILGLHV         1514
StPV_Cheravirus                 ------------------------MISGDCGVISFAPGGSTLEGSGVVPPKVCIM---HD         33
P_Stephan                       ---------KY-VC-SPGIGYKGHFGAGDCGVVLFSPTKV------GQPPLVCIM---HD         1427
P12_cheravirus                  ---------KY-IC-SPGIGYKGHFGAGDCGVVLFTPTKV------GQPPLVCIM---HD         1576
P3_cheravirus                   ---------KY-IC-SPGIGYKGHFGAGDCGVVLFTPTKV------GQPPLVCVM---HD         1573
P4_cheravirus                   ---------KY-IC-SPGIGYKGHFGAGDCGVVLFTPTKV------GQPPLVCVM---HD         1523
                                                             *                        :
```

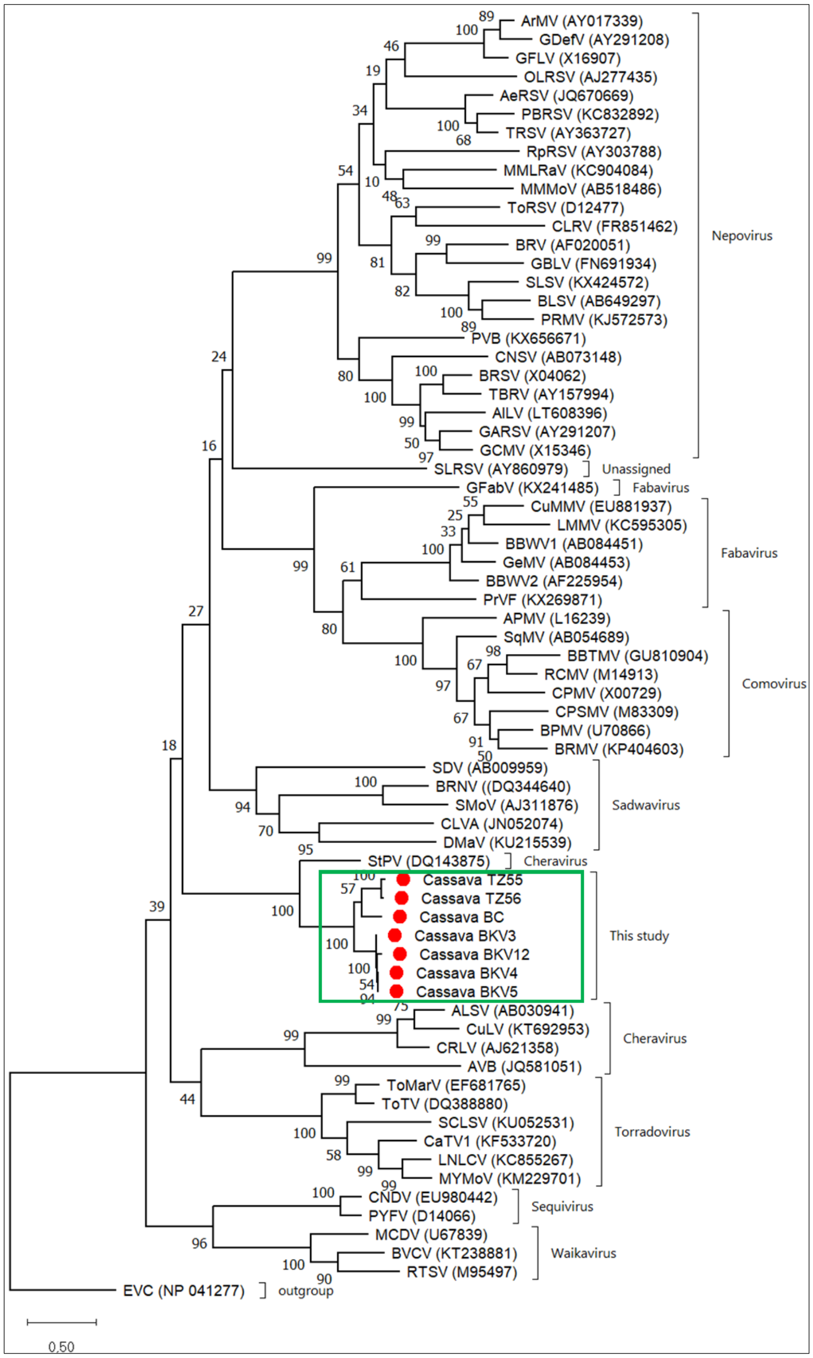

Figure S2. Phylogenetic analysis of the coat protein amino acid for the five isolates detected in cassava (strong green triangles and green frame) and of type isolates from recognized species in the family *Secoviridae* (detailed information on these viruses is provided in supplementary material 4). For each *Secoviridae* species, the amino acid sequence of the CP(s) was deduced from the nucleotide sequence of the corresponding genomic RNA from the type isolate. The alignment was generated using ClustalW integrated into MEGA X. The Maximum likelihood phylogenetic tree was also reconstructed in MEGA X using the Poisson model with uniform distribution for amino acid sequence alignments. Bootstrap values are indicated at the main branch nodes (1000 replicates). The bar represents the number of amino acid substitutions per site. The tree was rooted using the combined sequence of the three CPs from poliovirus (EVC, species Enterovirus C, genus Enterovirus, family *Picornaviridae*).

Figure S3. Multiple amino acid sequence alignment of the conserved “CG-GDD” motif shows the insertion of 36 amino acids (highlighted in red) upstream of the “GDD” motif in the polymerase domain**.**

```
ArMV_subgroup_A_nepovirus  YRE------------------------------------------CFDRCVVLITYGDD        1838
ToRSV_subgroup_C_nepovirus LVN------------------------------------------NFKQEVCLIVYGDD        1875
CsTVL_Torradovirus         SLR----------------------------------SFTVDCTSSDFERLFVAVYGDD        1523
SMoV_sadwavirus            DPG-------------------------------------LVPYRVMRSHCTFSVYGDD        1615
CPMV_comovirus             QAP-------------------------------------ELMVQSFDKLIGLVTYGDD        1546
CuLV_(Cheravirus)          NDM------------------------------------DLYPLYSFKQLVSYAVYGDD        1855
ALSV_Cheravirus            VHK------------------------------------ALYPLYSFRTLVSYAVYGDD        1770
CRLV_Cheravirus            ELN------------------------------------SLYPLHSFRQMVAYATYGDD        1943
AVB_(Cheravirus)           LKS----------------------------------------HFWFTKNVAFAVYGDD        1868
StPV_(Cheravirus)          LGE------------------------------------AFVHRSIMDREVHFAVYGDD        471
P_Stephan                  LYREASIIALVEERVRYLKDFLQDDDALILQKARQIVTQPMVSKSVMDDSVTIAVYGDD        1895
P12_cheravirus             LRTEASVVALAEEKFRYLRDFSKEDDDFLRKKAFTIVTQPMVSKELMDSNVAIAVYGDD        2044
P3_cheravirus              LRTEASIVALAEERFKYLRDFSKEDEELLRKRAFTIVTQPMVSKEIMDSNVAIAVYGDD        2041
P4_cheravirus              LRTEASVVALAEERFKYLRDFSKEDEELLRSRAFTIVTQPMVSKKLMDSNVAIAVYGDD        1991
```

Figure S4. Phylogenetic trees reconstructed using **(A)** the amino acid sequences of the “Pro-Pol” region, **(B)** the amino acid sequences of the entire P1 polyprotein, **(C)** the complete/nearly nucleotide sequence of the RNA1 segment **(D)** the amino acid sequences of the coat protein block, **(E)** the amino acid sequences of the entire P2 polyprotein and **(F)** the nearly/complete nucleotide sequence of the RNA2 segment for the new *Secoviridae* members described in this study. The alignment was generated using ClustalW integrated into MEGA X using the Poisson model for amino acids and the GTR+GI model for nucleotide sequences. The neighbour-joining method was used for phylogenetic reconstruction in Mega X. Bootstrap values are indicated at the main branch nodes (1000 replicates). The bar represents the number of amino acid substitutions per site.

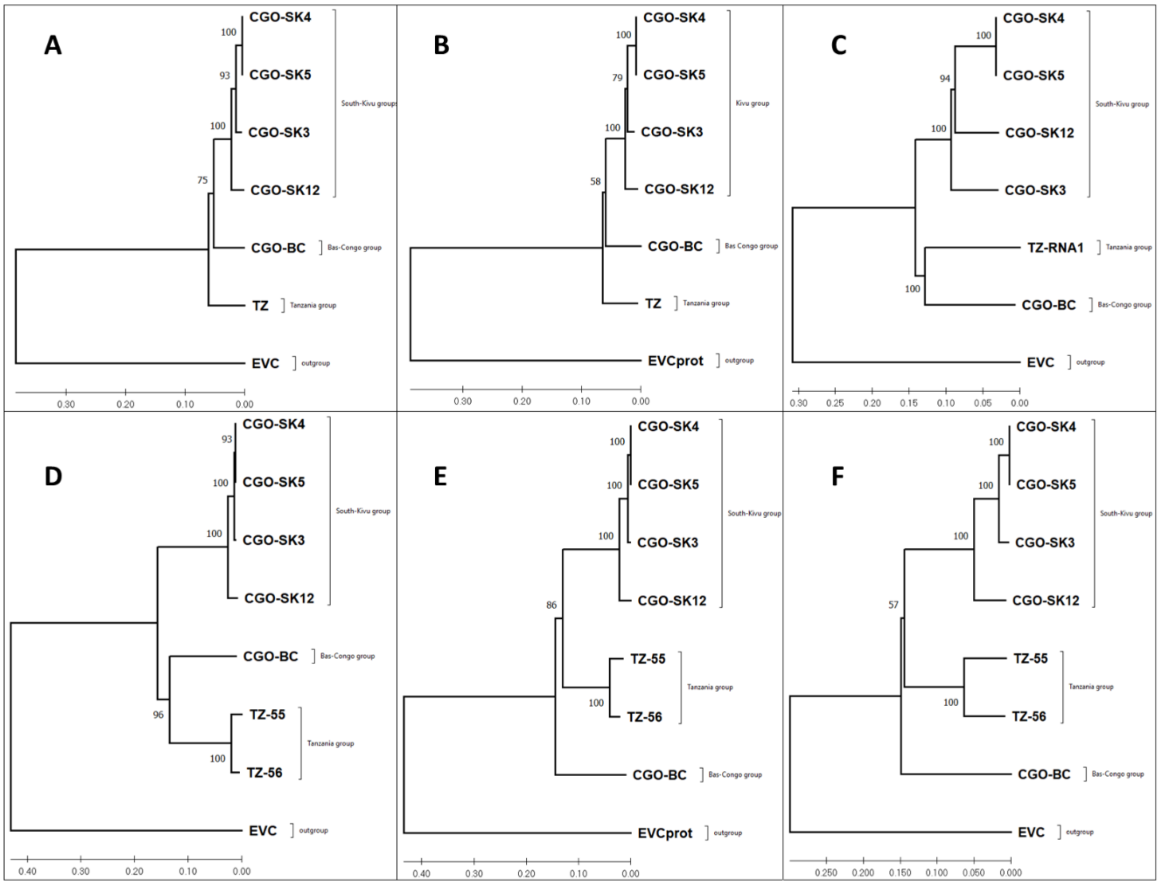

Figure S5: Multiple amino acid sequence alignment of the C-terminal region of the polymerase domain of viruses reported in this study, together with the arracacha virus A. The figure shows a putative cleavage site that could delineate a putative domain downstream of the polymerase.

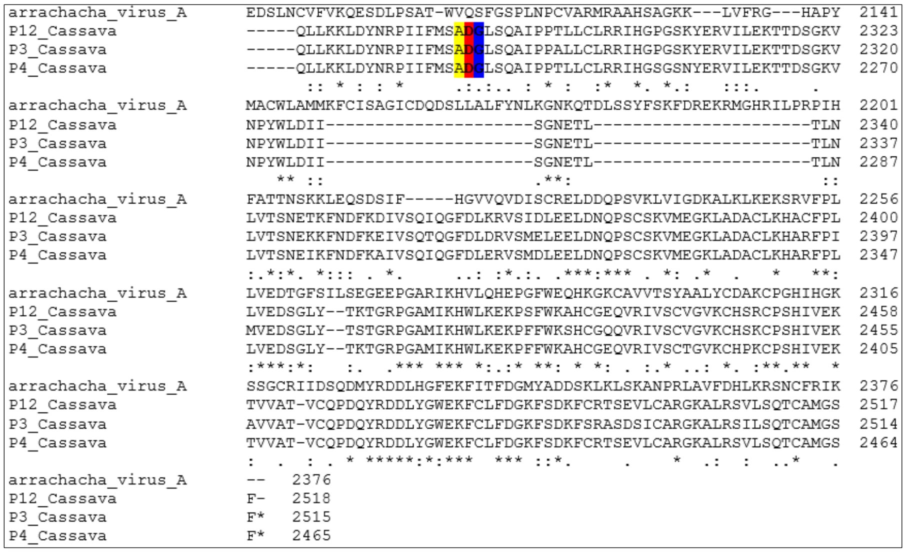

Figure S6: Multiple amino acid sequence alignment showing conserved sites of ITPases from representative members of various kingdoms of life. The alignment was built using Mview (Brown, Leroy, and Sander 1998)

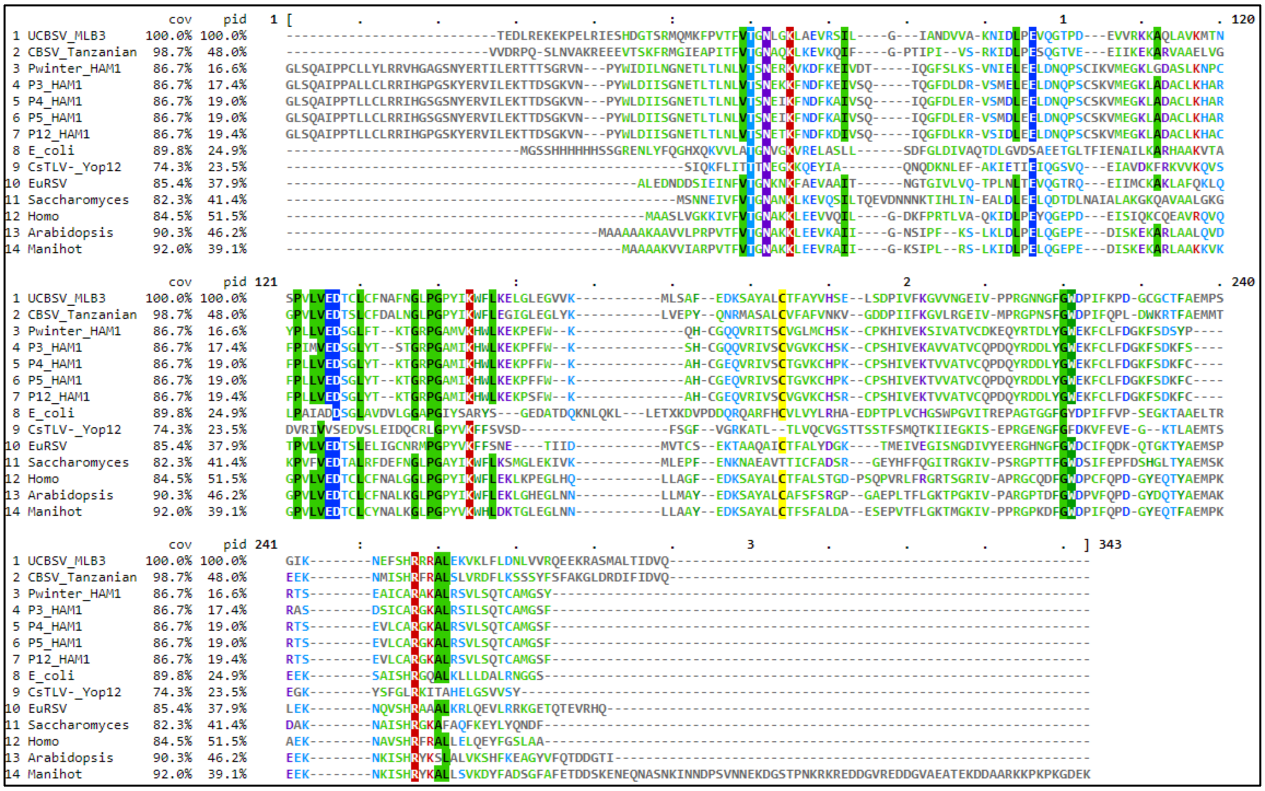

Supplement: Supplementary file 1 [file viruses-18-00084-s001.zip › Supplementary material.pdf]
